# Differential Leukocyte MicroRNA Responses Following Pan T Cell, Allorecognition and Allosecretome-Based Therapeutic Activation

**DOI:** 10.1007/s00005-021-00634-5

**Published:** 2021-10-22

**Authors:** Xining Yang, Wendy M. Toyofuku, Mark D. Scott

**Affiliations:** 1 Terry Fox Laboratory, BC Cancer, Vancouver, BC V5Z 1L3 Canada; 2 Department of Pathology and Laboratory Medicine, University of British Columbia, Vancouver, BC V6T 1Z3 Canada; 3 University of British Columbia Centre for Blood Research, Vancouver, BC V6T 1Z3 Canada; 4 Canadian Blood Services and the Centre for Blood Research, Life Sciences Centre, University of British Columbia, 2350 Health Sciences Mall, Vancouver, BC V6T 1Z3 Canada

**Keywords:** T lymphocyte, Immunomodulation, miRNA, Alloactivation, Secretome, Therapeutics

## Abstract

Effective immunomodulation of T-cell responses is critical in treating both autoimmune diseases and cancer. Our previous studies have demonstrated that secretomes derived from control or methoxypolyethylene glycol mixed lymphocyte alloactivation assays exerted potent immunomodulatory activity that was mediated by microRNAs (miRNA). The immunomodulatory effects of biomanufactured miRNA-based allo-secretome therapeutics (SYN, TA1, IA1 and IA2) were compared to Pan T-cell activators (PHA and anti-CD3/CD28) and lymphocyte alloactivation. The differential effects of these activation strategies on resting peripheral blood mononuclear cells (PBMC) were assessed via T-cell proliferation, subset analysis and miRNA expression profiles. Mitogen-induced PBMC proliferation (> 85%) significantly exceeded that arising from either allostimulation (~ 30%) or the pro-inflammatory IA1 secretome product (~ 12%). Consequent to stimulation, the ratio of CD4 to CD8 cells of the resting PBMC (CD4:CD8; 1.7 ± 0.1) decreased in the Pan T cell, allrecognition and IA1 activated cells (averages of 1.1 ± 0.2; 1.2 ± 0.1 and 1.0 ± 0.1). These changes arose consequent to the expansion of both CD4^+^CD8^+^ and CD4^–^CD8^–^ populations as well as the shrinkage of the CD4 subset and the expansion of the CD8 T cells. Importantly, these activation strategies induced vastly different miRNA expression profiles which were associated with significant differences in cellular differentiation and biological function. These findings support the concept that the “differential patterns of miRNA expression” regulate the biologic immune response in a “lock and key” manner. The biomanufacturing of miRNA-enriched secretome biotherapeutics may be a successful therapeutic approach for the systemic treatment of autoimmune diseases (TA1) and cancer (IA1).

**Supplementary Information:**

The online version contains supplementary material available at 10.1007/s00005-021-00634-5.

## Introduction

Effective modulation of the T-cell immune response is critical in treating both autoimmune (e.g., multiple sclerosis, arthritis, type I diabetes) and immunodeficient (e.g., cancer) diseases (Liebman 2016; Naidoo et al. 2014; Wang et al. 2015; Yang et al. 2019). Treatment of autoimmune disease has commonly focused on cytotoxic agents (e.g., cyclosporine) to remove self-reactive immune cells and more recently, cytokine absorptive approaches (e.g., etanercept) (Fathman and Myers 1992; Feutren 1992; Khanna et al. 2003; Scott 2014). These “anti-inflammatory” approaches have been highly successful in preventing graft rejection and in modulating disease severity in a number of autoimmune diseases. In contrast, in diseases characterized by a weak immune response (e.g., cancer), enhancement of the inflammatory response have attempted to use mitogens (e.g., phytohemagglutinin: PHA), monoclonal antibodies (mAb; e.g., anti-CD3/CD28), cytokines (e.g., IL-2) and, less commonly, induction of an alloresponse (e.g., Coley’s toxins and graft versus leukemia effects) to strengthen the immune response (Barrett 1997; Barrett and Childs 2000; Deng et al. 2001; Fabre 2001; Liao et al. 2011; Mire-Sluis et al. 1987; Starnes 1992a,b; Stathopoulos et al. 2008; Trickett and Kwan 2003). However, these pro-inflammatory approaches have been much less successful due to overly robust responses resulting in adverse events such as cell injury and cytokine release syndrome.

In contrast to these traditional approaches, bioengineering of cell surfaces have also been shown to induce an immunomodulatory effect. The immunocamouflage of the lymphocyte membrane by the covalent grafting of biocompatible polymers (e.g., methoxypolyethylene glycol: mPEG) has been demonstrated to induce a pro-tolerogenic environment both in vitro and in vivo (Kang et al. 2017; Kyluik-Price et al. 2014; Kyluik-Price and Scott 2016; Le and Scott 2010; Murad et al. 1999; Wang et al. 2011). Surprisingly, these studies also found that the secretome from the control and mPEG allorecognition-based mixed lymphocyte reactions (MLR) also exerted potent immunomodulatory activity that was mediated by microRNAs (miRNA) (Scott et al. 2019; Wang et al. 2011, 2015; Yang et al. 2019).

Mammalian miRNA are short (~ 22-nucleotide long) RNA molecules that regulate messenger RNA (mRNA) expression at a posttranscriptional level. Currently, more than 2,000 miRNA have been identified in humans (Hammond 2015). Since their discovery in 1993 in the nematode *Caenorhabditis elegans*, the role of miRNA has transitioned from being “junk nucleic acids” to being recognized as key regulators of a multitude of biological processes including the immune response (Grigoryev et al. 2011; Neilson et al. 2007). However, to date, the vast majority of research into the role of miRNA in immune response has been largely observational, with specific miRNA being used as biomarkers of immunological and/or pathogenic disease states (Cortez and Calin 2009; Hayes et al. 2014). Indeed, the profiles of cellular miRNA expression can actively reflect the systemic alterations in immune activity (Dudda et al. 2013; Rossi et al. 2011; Teteloshvili et al. 2015). More recently, due to their rapid response and sensitivity to the changing cellular environment, miRNA have also been used as potential biomarkers for drug efficacy prediction and therapeutic approaches (Hayes et al. 2014).

However, despite their vast potential as biomarkers, relatively little research has been done on miRNA as therapeutic agents largely due to the complexity of miRNA-based bioregulation (Bartel 2009, 2018). It is important to note that miRNA are “low fidelity” in nature in that a single miRNA can interact with potentially hundreds of genes and a single gene can be regulated by hundreds of miRNA (Grimson et al. 2007; Shukla et al. 2011). Hence, replicating the “pattern of miRNA expression” is key to exerting a desired bioregulatory effect. To reproduce the miRNA patterns necessary to induce either a pro-inflammatory or tolerogenic immune response, our laboratory has utilized an alloresponse-based biomanufacturing approach. In this strategy, alloreaction-derived cell-free secretomes are manufactured from resting peripheral blood mononuclear cells (PBMC) or control- or mPEG-MLR (Scott et al. 2019; Wang et al. 2011, 2015; Yang et al. 2019). As previously demonstrated, the miRNA-based secretomes systemically re-orientate the immune system towards either a pro-inflammatory (IA1) or pro-tolerogenic (TA1) state (Scott et al. 2019; Wang et al. 2015; Yang et al. 2019). Importantly, miRNA prepared from resting immune cells (human PBMC or murine splenocytes) exhibited minimal immunomodulatory activity (Scott et al. 2019; Yang et al. 2019).

In this study, we further defined the differential effects of our previously described miRNA-based secretome therapeutic approach (Scott et al. 2019; Wang et al. 2015; Yang et al. 2019) to pan T cell (PHA; anti-CD3/CD28) and allorecognition-based activation of resting human PBMC. T-cell differentiation as well as the differential miRNA production induced by the different activation strategies was assessed. Within the miRNA, we further analyzed a panel of thirteen differentially expressed miRNA to associate the miRNA “pattern of expression” with the PBMC differentiation and biological activity. As will be demonstrated, highly distinct intracellular miRNA expression patterns were observed between the Pan T-cell activators, allorecognition and secretome therapeutics. Indeed, these studies support the view that a “lock and key” mechanism based on the “pattern of miRNA expression”, rather than changes in a single miRNA, underlie the biologic signaling necessary to induce a desired (tolerance versus inflammation) immunomodulatory response.

## Methods and Materials

### Human PBMC

All human experiments were done in accordance with the University of British Columbia Clinical Research Ethics Board and the Code of Ethics of the World Medical Association (Declaration of Helsinki). The PBMC were isolated from donor whole blood using Histopaque-1077 (Sigma-Aldrich, St. Louis, MO, USA) as described before (Kang et al. 2017; Wang et al. 2011, 2015; Yang et al. 2019). Human PBMC were washed and resuspended in AIM V media (research grade; ThermoFisher Scientific, Grand Island, NY, USA).

### Secretome Production

Acellular secretome products IA1, IA2, TA1 and SYN were manufactured as previously described (Yang et al. 2019). IA1 and TA1 were manufactured from around 20 unique donor PBMC combinations due to their strong allorecognition response. IA2 and SYN were produced from random single donor from this donor pool. Then secretome products were tested on recipient PBMC that were both related and unrelated to the donor PBMC. Technical duplicates were done for each batch of secretome product per experiment, and as least three independent experiments were done for each type of secretome product on independent recipient PBMC.

### Differential Effects of Activation Strategies on Resting Leukocytes

**Fig. 1:**
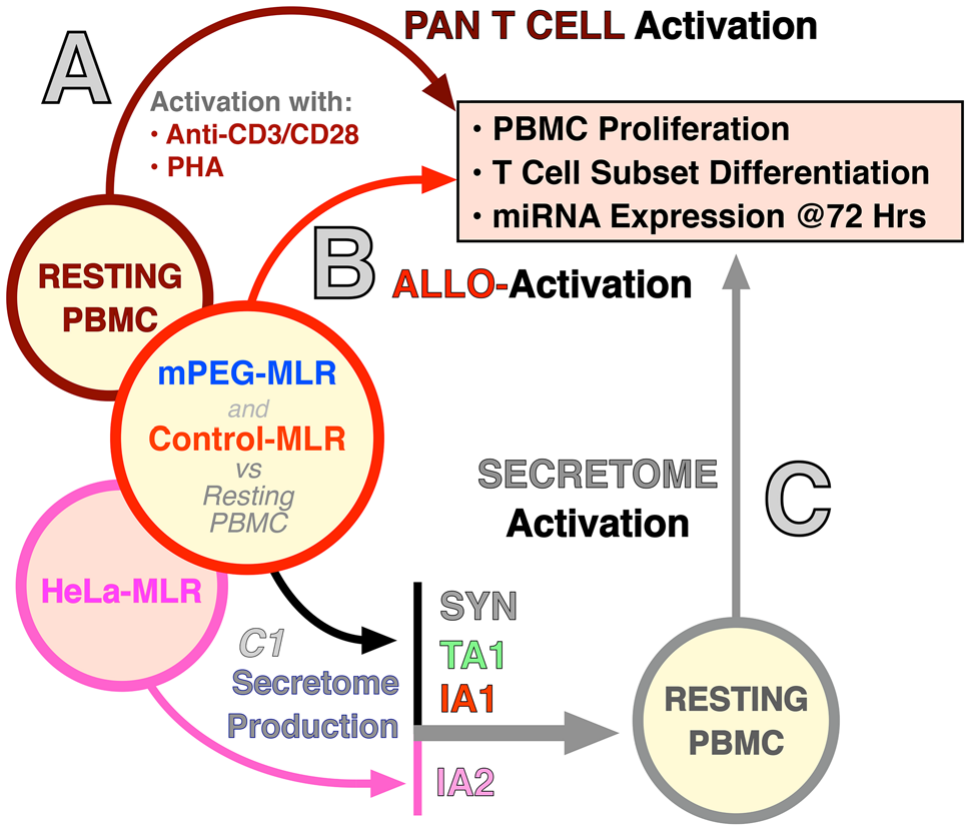
Schematic presentation of the three differential activation strategies (**A**–**C** Pan T; Allorecognition; and Secretome) of PBMC activation studied. T-cell proliferation and subset differentiation were measured via flow cytometry and leukocyte miRNA expression levels which were compared to untreated resting PBMC. Pan T-cell activation (**A**) of freshly isolated human resting PBMC utilized anti-CD3/anti-CD28 or PHA. Alloactivation (**B**) was conducted via an MLR with or without mPEG (20 kDa mPEG; grafting concentration of 2 mM) immunocamouflage of one MHC-disparate donor. The negative control consisted of the untreated resting PBMC. Secretome activation consisted of two phases: production of the secretome therapeutics ***(C1)*** and the activation (**C**) of freshly collected donor PBMC with the manufactured secretome therapeutic. The SYN, TA1 and IA1 was derived from resting PBMC, mPEG-MLR and control MLR, respectively. The IA2 secretome product was produced using an PBMC-cancer (HeLa) cell MLR model (HeLa-MLR). Proliferation and subset analysis of the different activation strategies were optimized as previously described (Yang et al. 2019). miRNA profiling of the activated PBMC of the differential strategies (**A**–**C**) was done at 72 h and compared to untreated resting PBMC

The effects of Pan T-cell activators [i.e., anti-CD3/anti-CD28 and mitogen (phytohemagglutinin: PHA)], alloactivators (i.e., control MLR and camouflaged MLR) and secretomes (i.e., SYN, TA1, IA1 and IA2) on the activation of resting leukocytes were compared (Fig. 1). In Pan T-cell activation, freshly isolated human PBMC were stimulated with anti-human CD3e in the presence of soluble anti-human CD28 for 3 days, or with PHA for 4 days as previously described (Fig. 1A) (Yang et al. 2019). Alloactivation was conducted in an MLR system with or without succinimidyl valerate activated mPEG (Laysan Bio Inc. Arab, AL) for 10 days (Fig. 1B) (Yang et al. 2019). Effects of alloactivation were compared to untreated resting PBMC. To explore the immunomodulatory effects of alloactivation-secretome-derived therapeutics, cell-free TA1 and IA1 biologics were produced from mPEG-MLR and MLR respectively. Cell secretions from untreated resting PBMC were collected as SYN (Yang et al. 2019). A lymphocyte-cancer (HeLa) cell biotherapeutic IA2 was concurrently developed from a HeLa-MLR as previously described (Yang et al. 2019). In allo- and secretome activation studies, proliferation and phenotyping of treated PBMC were measured at day 10 (Fig. 1C). For all activation strategies, PBMC miRNA expression profiles were measured at 72-h post-treatment.

### T-Cell Proliferation and Phenotyping

As documented in our previous publications, systemic T-cell immune responses encompass a broad range of T-cell subpopulations that give rise to a “net” inflammatory or tolerogenic environment at both the local and systemic level (Kang et al. 2017; Kyluik-Price et al. 2014; Kyluik-Price and Scott 2016; Scott et al. 2019; Wang et al. 2012, 2015; Yang et al. 2019). In this study, T-cell lymphocyte CD3^+^CD4^+/–^ and CD3^+^CD8^+/–^ subpopulations were measured using fluorescently labeled anti-CD3, CD4 and CD8 monoclonal antibodies (mAb; BD Pharmingen, San Jose, CA, USA). All samples were acquired using the FACSCalibur flow cytometer and CellQuest Pro software (BD Biosciences, San Jose, CA, USA) for both acquisition and analysis.

### Leukocyte miRNA Expression

Total RNA was extracted from resting PBMC ± treatment (anti-CD3/anti-CD28, PHA, MLR ± mPEG, SYN, IA1, IA2 and TA1) following 72 h incubation using the mirVana™ PARIS™ kit (Ambion, Life Technologies, Grand Island, NY, USA). Following processing, the highly enriched small RNA fraction containing miRNA was prepared using RNase/DNase free water. To partially characterize and quantify the relative abundance the miRNA species present in the resting and differentially activated PBMC, quantitative reverse transcription polymerase chain reaction (qRT-PCR) was done using the miScript miRNA PCR Array system (Qiagen, Frederick, MD, USA) for the human immunopathology pathway as described before (Wang et al. 2015; Yang et al. 2019). These miRNA microarrays plates were run using an Applied Biosystems StepOnePlusTM Real Time PCR System (ThermoFisher Scientific, Grand Island, NY, USA). This human immunopathology array plate is pre-configured with the appropriate RNA and quality controls and has been validated by Qiagen. This array profiles the expression of 84 miRNA differentially expressed during normal and pathological immune responses. It is worth noting that the 84 miRNA examined are not all inclusive and that other miRNA are likely to be present and could be of immunoregulatory importance. Threshold and baseline were defined and the resultant threshold cycle (Ct) values were calculated using the StepOnePlus software (v.2.1). Ct values were exported and analyzed using the Qiagen GeneGlobe Online Analysis Center using the Relative Quantification qRT-PCR method for analysis (ΔΔCt). The data shown represent three biological replicates analyzed independently by qRT-PCR. The data shown represent three biological replicates analyzed independently by qRT-PCR.

### Statistical Analysis

All data were expressed as mean ± standard error mean (SEM). A minimum of three independent experiments were performed in duplicates for all studies. Data analysis was conducted using GraphPad Prism 6.0 (GraphPad Software, Inc., San Diego, CA, USA). For comparison of means, a one-way analysis of variance (ANOVA) was performed. When significant differences were found, a post hoc Tukey test was conducted for pair-wise comparison of means. For significance, a minimum p < 0.05 (labeled with “*”) was used. The symbols “**”, “***” and “****” were used for labeling *p* value of < 0.01, < 0.001 and < 0.0001, respectively.

## Results

**Fig. 2:**
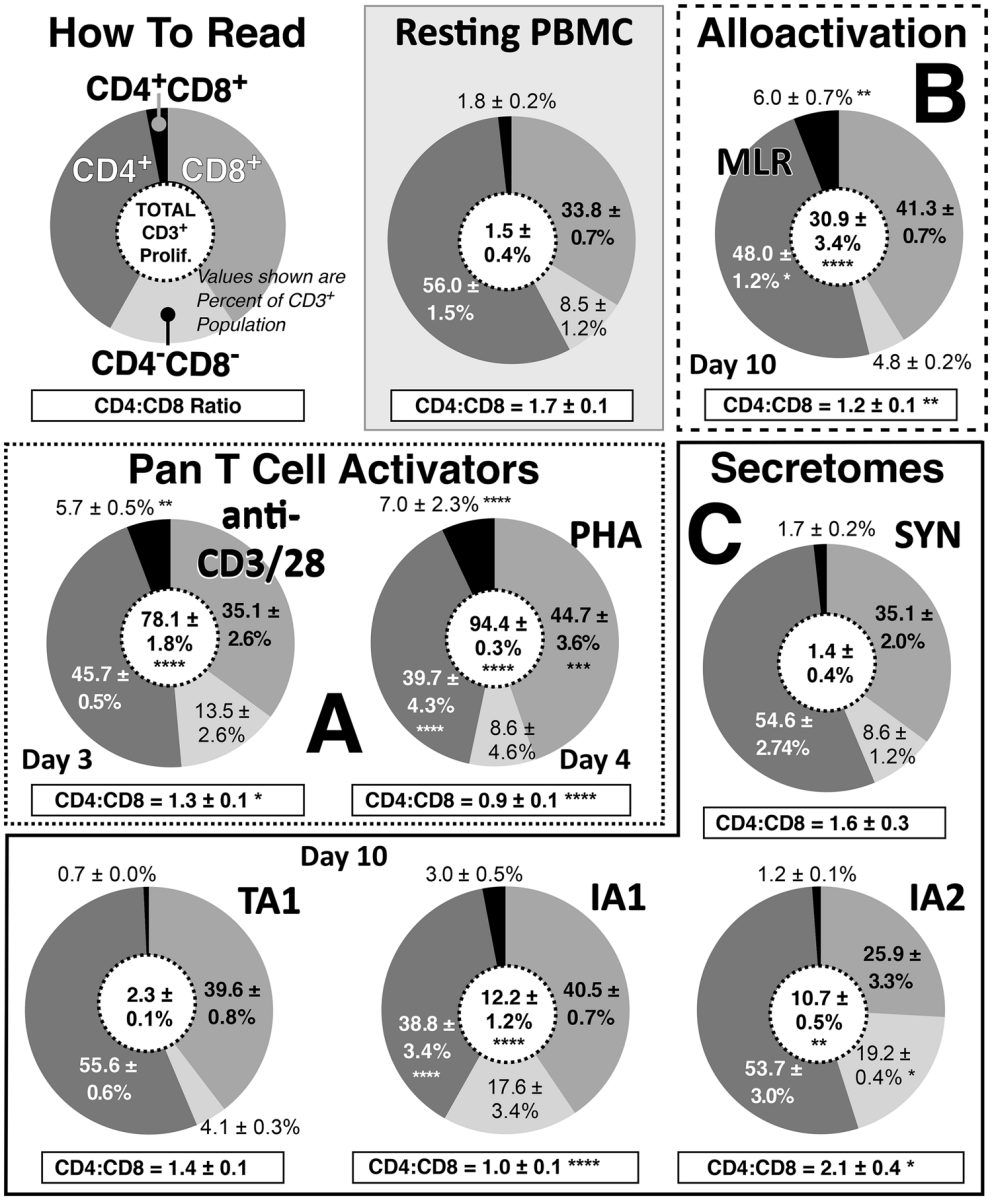
Effects of the different activation strategies on T-cell CD4^±^ and CD8^±^ subset differentiation. Upper left panel describes the data presentation. Briefly the centre circle indicates total CD3^+^ proliferation (Yang et al. 2019) and the slices of the pie-chart denote percentages of the indicated CD4/CD8 subsets of the total CD3^+^ cells at the indicated day. Resting CD3^+^ PBMC are shown for comparison at 10 days’ incubation. **A** Results of the Pan T-cell activators Anti-CD3/CD28 at Day 3 and PHA at Day 4. **B** Control MLR alloactivation at Day 10. **C** Secretome therapeutics at Day 10 post activation. Values shown are mean ± SEM. Statistics (all relative to resting PBMC): *p < 0.05, **p < 0.01, ***p < 0.001, ****p < 0.0001, *n* ≥ 4

miRNA-based secretome therapeutics (SYN, TA1, IA1 and IA2) have been successfully produced in our laboratory based on allorecognition (± polymer-mediated immunocamouflage) based MLR (Scott et al. 2019; Wang et al. 2015; Yang et al. 2019). As demonstrated in our previous studies, the effects of our acellular secretome products to Pan T-cell activators (PHA or anti-CD3/CD28) on CD3^+^ T-cell proliferation (Fig. 2, center circle) as well as T regulatory (CD4^+^Foxp3^+^CD25^+^) and Th17 (CD4^+^IL17^+^) cells were distinct (Scott et al. 2019; Wang et al. 2015; Yang et al. 2019) To further define the proliferation pattern within the CD3^+^ cells, CD4^±^ and CD8^±^ subsets and the CD:CD8 ratio were further analyzed (Fig. 2). As shown, resting PBMC demonstrated minimal proliferation and a CD4:CD8 ratio of 1.7 ± 0.1. In contrast, the Pan T-cell activators anti-CD3/CD28 and PHA induced massive CD3^+^ cell proliferation and altered the CD4:CD8 ratio (Fig. 2A). Despite both PHA and anti-CD3/CD28 being Pan T-cell activators, there were differences in how these agents modulated the CD4/CD8 differentiation. PHA, but not anti-CD3/CD28, significantly increased the CD8^+^ population while simultaneously decreasing the CD4^+^ population resulting in a significant (*p* < 0.0001) decrease of CD4:CD8 ratio relative to resting PBMC (1.7 to 0.9). mAb activation also decreased the ratio but not as dramatically as PHA. Alloactivation, in comparison to the highly potent Pan T-cell activation, induced a more moderate proliferation of CD3^+^ cells relative to resting PBMC (Scott et al. 2019; Wang et al. 2015; Yang et al. 2019) The reduction in proliferation arose consequent to < 10% of T cells within a population typically being capable of allorecognition (Fig. 2B) (Abul et al. 2015; Nisbet et al. 1969). Alloactivation similarly decreased (*p* < 0.01) the CD4:CD8 ratio.

The secretome products showed significant variability (at Day 10) in their effects when used to activate resting PBMC. As expected, resting PBMC treated with the SYN secretome were virtually identical to the resting PBMC with regards to proliferation, subset analysis and CD4:CD8 ratio. Similarly, the tolerogenic TA1 preparation induced minimal proliferation and no substantive changes in the subset differentiation or the CD4:CD8 ratio. Of note, the small increase in CD3^+^ proliferation (2.3 ± 0.1%) supports earlier observations showing increased proliferation/differentiation of regulatory T cells (Treg; CD4^+^Foxp3^+^) in TA1-treated PBMC (Kang et al. 2017). However, in contrast to the SYN and TA1 products, IA1 and IA2 showed significant variation from both the resting PBMC and from each other (Fig. 2C). IA1, derived from a control MLR, significantly increased the total CD3^+^ cell proliferation and decreased the relative abundance of CD4^+^ cells (38.8 ± 3.4% versus 56.0 ± 1.5% for resting PBMC). Consequent to this change, the CD4:CD8 ratio was significantly reduced (1.0 ± 0.1; *p* < 0.001) suggestive of a pro-inflammatory state and similar to that noted with the Pan T-cell activators. Interestingly, the cancer cell (HeLa) stimulated biologic IA2, while inducing a similar level of CD3^+^ cell proliferation, showed dramatically different phenotype distribution (Fig. 2C). In contrast to IA1, which reduced the CD4:CD8 ratio, IA2 significantly increased the ratio relative to both IA1 and the resting PBMC (2.1 ± 0.4, 1.0 ± 0.1, and 1.7 ± 0.1; respectively) consequent to a decrease in CD8^+^ cells. These findings further support our previous study suggesting that the anti-HeLa effects of IA2 treated PBMC were distinct from that of IA1 treated PBMC (Scott et al. 2019; Yang et al. 2019).

Interestingly, both IA1 and IA2 activation also resulted in a significant expansion (*p* < 0.05) of double negative (CD4^–^CD8^–^) T cells. These double negative cells, while poorly understood, have been implicated in both inflammation and as regulatory cells (D’Acquisto and Crompton 2011). Under certain activating stimuli, CD4^–^CD8^–^ T cells can display the phenotype of effector cells that are capable of producing pro-inflammatory cytokines e.g., IFN-γ, IL-17A, and the phenotype of suppressor cells that secrete immune regulatory cytokine (e.g., IL-10). Consequently, CD4^–^CD8^–^ T cells have been described in promoting neuroinflammation after ischemic stroke (Meng et al. 2019), as well as preventing autoimmune diseases and graft-versus-host disease (GvHD) (Brandt and Hedrich 2018; Ford et al. 2007; Lev et al. 2012; Young et al. 2003). Interestingly, a recent study revealed the potential role of CD4^–^CD8^–^ T cells in limiting alloreactive immune response by suppressing the CD4 T-cell functionality (Haug et al. 2019). Surprisingly perhaps, TA1 treatment tended to reduce the CD4^–^CD8^–^ population despite previous research documenting its potent tolerogenic activity in a murine model of Type 1 diabete (Wang et al. 2015).

In contrast, double-positive (CD4^+^CD8^+^) T cells were reduced in the TA1, IA1 and IA2 (0.7 ± 0.0, 3.0 ± 0.5, and 1.2 ± 0.1%, respectively) activated cells relative to Pan T-cell activation (PHA: 7.0 ± 2.3%; and anti-CD3: 5.7 ± 0.5%), and alloactivation (6.0 ± 0.7%). The biologic functions of CD4^+^CD8^+^ T cells remain unclear with, perhaps most, studies reporting a pro-inflammatory role in cancers though some evidence of an immunosuppressive role has been reported (Bohner et al. 2019; Overgaard et al. 2015; Parel and Chizzolini 2004; Rahemtullah et al. 2006). CD4^+^CD8^+^ T cells are increased in urologic cancers patients and found to be high type-2 cytokine producers favoring a Th2 response in vitro while inhibiting Th1 cells known to play a crucial role in anti-tumor immunity (Bohner et al. 2019). Hence, the decrease in the CD4^+^CD8^+^ cells post secretome (TA1, IA1 and IA2) activation, relative to pan T cell or alloactivation, may have clinical benefit and be indicative of the secretomes’ more “restrained” immunoactivation.

**Fig. 3:**
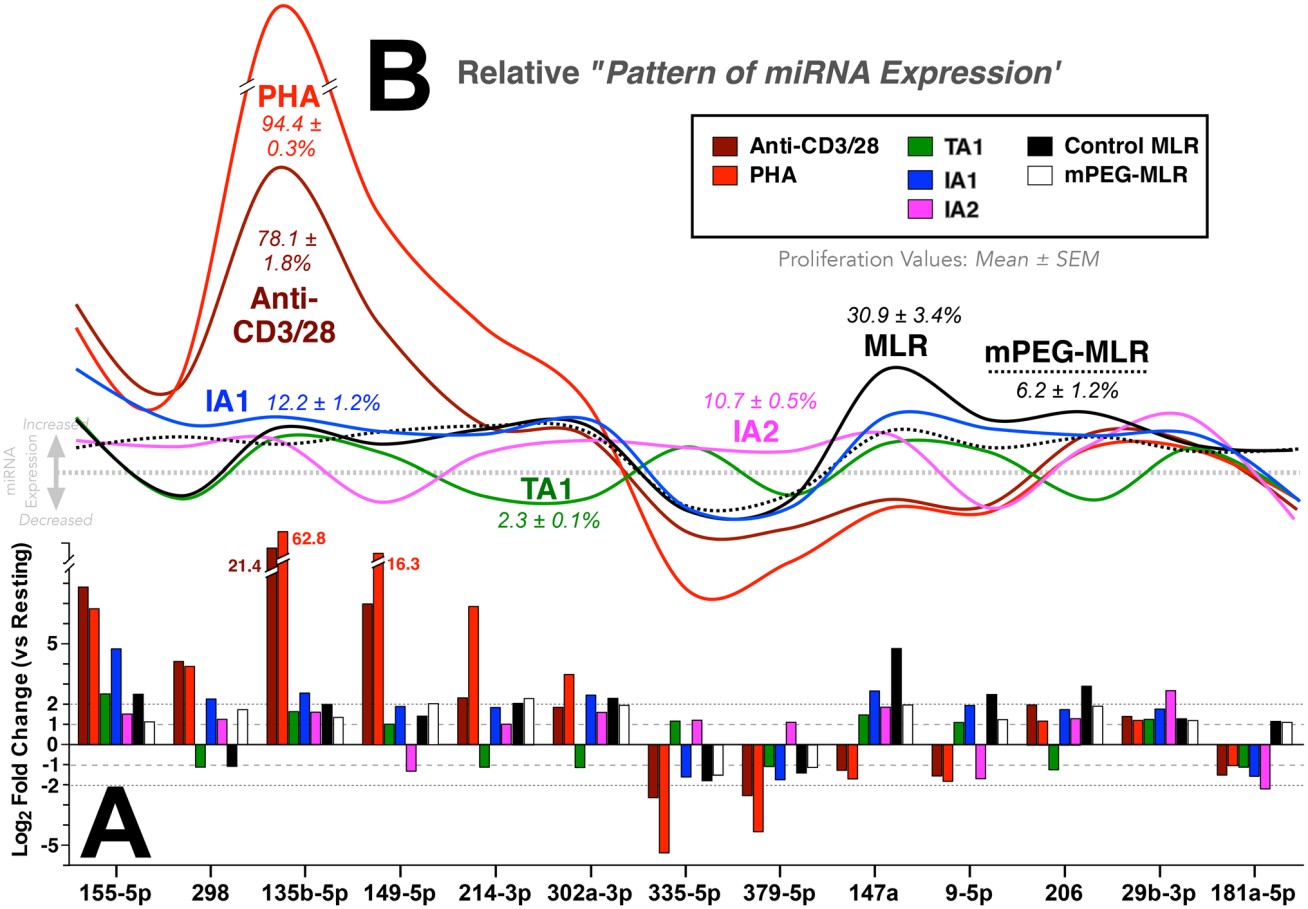
Significantly disparate miRNA expression patterns arise consequent to the different PBMC activation strategies. **A** Log_2_ fold change analysis similarly demonstrated the differential effects of Pan T cell activators, secretome therapeutics and allorecognition (control- and mPEG-MLR) on the expression of thirteen-selected miRNA relative to resting PBMC (solid line at 0). Grey dashed lines of − 2, − 1, 1 and 2 values are shown for comparison purposes. Pan T-cell activators altered the PBMC miRNA expression to a much larger magnitude than the pro-inflammatory IA1 secretome or the control MLR. The SYN, pro-tolerogenic TA1 and cancer-derived IA2 secretome exerted significantly different effects on miRNA expression as would be expected based on their differential effects on cell proliferation and CD4/CD8 differentiation. **B** The relative “pattern of miRNA expression” and percent proliferation of the different activation protocols from **A**. As shown, significant differences in the pattern of expression are noted—even amongst the pro-inflammatory (PHA, anti-CD3/28, IA1 and IA2) activators. The putative/described functions of these miRNA in biological studies are presented in Supplementary Table 1S

To determine how these differential T-cell activation strategies (i.e., Pan T-cell, allorecognition and secretome) affected resting human PBMC, the intracellular expression of 84 miRNA involved in immunopathology pathways were previously assessed (Scott et al. 2019; Wang et al. 2015; Yang et al. 2019). To further investigate the differential effects of the three T-cell activation strategies on resting PBMC, the “relative pattern of miRNA expression” was examined using a subset of thirteen differentially expressed miRNA [Fig. 3A; miRNA were selected via clustergram heatmap (Yang et al. 2019) and/or log_2_ fold change analysis]. The putative/described functions for these miRNA are summarized in Supplementary Table 1S (Scott et al. 2019; Wang et al. 2015; Yang et al. 2019). However, it is important to note that the “putative” functions of the distinct miRNA can vary significantly depending on the biological (e.g., prostate versus T cell) model used. Moreover, consequent to the low genetic fidelity of a single miRNA, we propose that the most informative approach is the analysis of the differential activation strategies on the relative “pattern of miRNA expression” across a number of miRNA (Fig. 3).

As shown, the Pan T-cell activators PHA and anti-CD3/CD28 demonstrated significant similarities in their “patterns of miRNA expression” and CD3^+^ proliferation (Fig. 3B). While these Pan T-cell activators are commonly used as activation surrogates for allorecognition and/or to enhance cytokine expression levels, comparison of the miRNA expression profiles show significant variances from the allorecognition expression profile (Control MLR; Fig. 3B). Surprisingly, minimal differences within the group of 13 miRNA were noted between the control- and mPEG-MLRs despite significant differences in T-cell proliferation. However, in the context of the overall 84 miRNA screened, distinct patterns are quite apparent (Supplemental Table 2S and Scott et al. (2019)). In contrast to both pan T cell activators and allorecognition, secretome (IA1, IA2 and TA1) activation gave rise to dramatically reduced levels of proliferation and subtler, though highly distinct, changes in miRNA expression (Fig. 3). The IA1 secretome (proliferation of 12.2 ± 1.2%) yielded a distinct miRNA expression profile from those of the Pan T-cell activators; though a muted similarity in the peaks and troughs was discernable. In contrast, the tolerogenic TA1 secretome-induced minimal proliferation (2.3 ± 0.1%) and produced a miRNA pattern that varied significantly from the pro-inflammatory and proliferation-inducing pan T cell and IA1 activators (Fig. 3). Indeed, previous clustergram heatmap analysis showed that miRNA expression induced by TA1 resembled resting and SYN treated PBMC but induced a potent Treg-mediated tolerogenic effect both in vitro and in vivo (Kang et al. 2017; Scott et al. 2019; Wang et al. 2015; Yang et al. 2019). Interestingly, the HeLa-PBMC manufactured IA2, while exerting a proliferative effect (10.7 ± 0.5%) similar to IA1, varied significantly from IA1 miRNA pattern (Fig. 3). Importantly, the differential patterns of expression (Fig. 3B) of the IA1, IA2 and TA1 miRNA were associated with differential biological effects on the naive PBMC. TA1 induced systemic tolerance (in vitro and in vivo) and IA1 enhanced PBMC-mediated inhibition of cancer cell growth while IA2 exhibited direct toxicity (apoptosis) of cancer cells (Wang et al. 2015; Yang et al. 2019). Importantly, the distinct expression patterns of miRNA between the Pan T-cell activators (PHA and anti-CD3/CD28), alloactivation the secretome products induced activation translated into dramatically different biological responses (Scott et al. 2019; Wang et al. 2015; Yang et al. 2019).

**Fig. 4:**
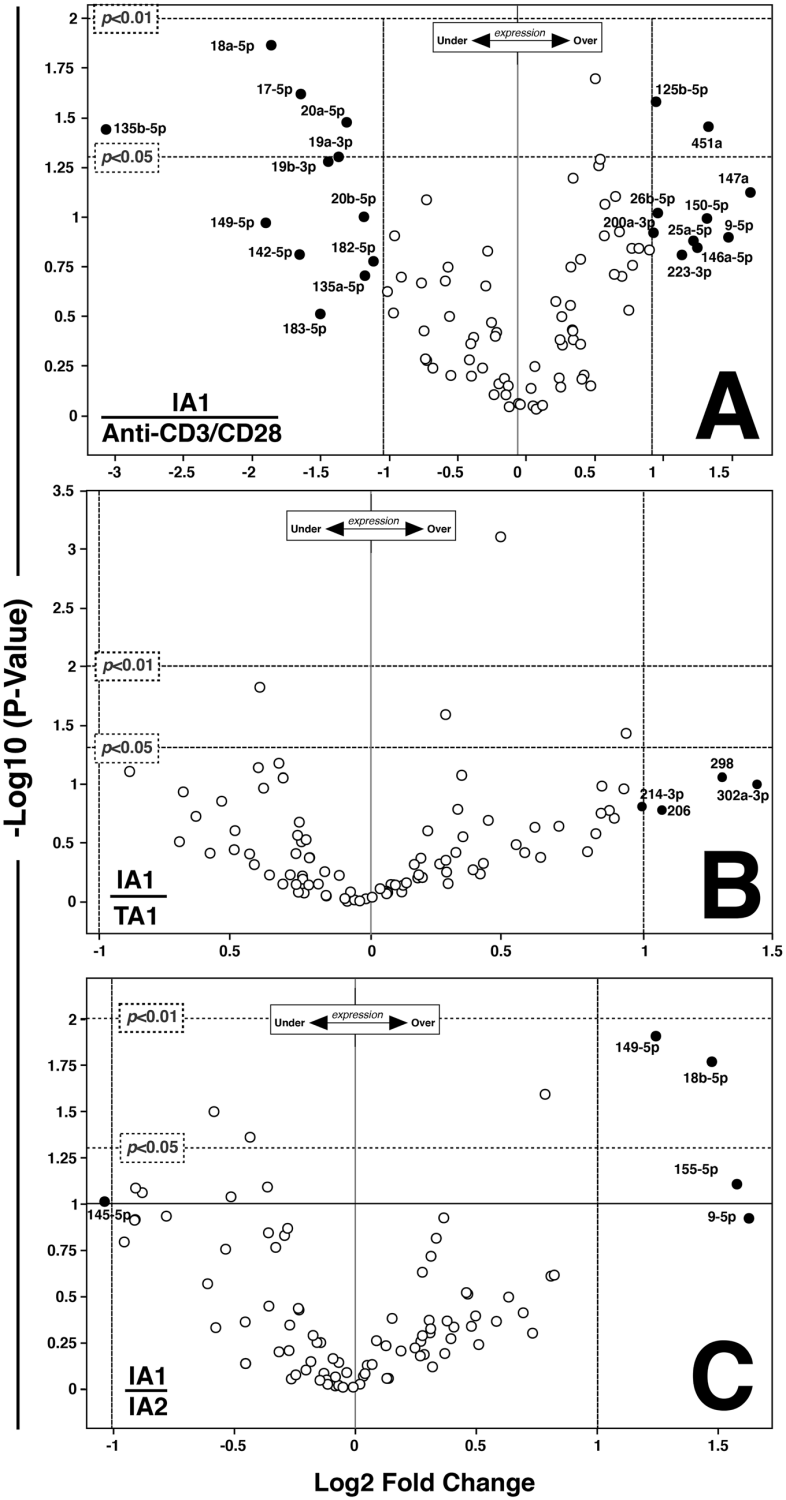
Volcano plot analysis of the secretome agents demonstrated differential PBMC miRNA expression profiles. Volcano plot analysis of selected secretome products was done to better compare the effects of these agents on resting PBMC miRNA expression at 72 h post activation (Yang et al. 2019). **A** IA1 versus anti-CD3/CD28. **B** IA1 versus TA1. **C** IA1 versus IA2. Vertical (Log_2_ Fold Change) and horizontal (− Log10; *p* value < 0.05 and 0.01) dashed lines denote miRNA over- or under-expressed. Unchanged (and unlabeled) miRNA are open circles while miRNA exhibiting significant changes are black circles and labeled

Consequent to our interests in using the IA1 secretome to activate resting PBMC to more efficiently kill cancer cells while preventing adverse events (e.g., cytokine storms), we more directly compared the miRNA expression profile of IA1 to pan T cell (anti-CD3/CD28; Fig. 4A), TA1 (Fig. 4B) and IA2 (Fig. 4C) activation via volcano plot analyses. Volcano plot analyses visualizes the differential miRNA data based on log scale changes and allows for statistical comparison of the expression of discreet miRNA between two samples (e.g., IA1 versus IA2)—but largely misses out on the overall PATTERN of changes seen with clustergram heatmaps (Yang et al. 2019) and Log2 fold change. As noted in Fig. 4A, distinct differences are noted between IA1 and anti-CD3/CD28. IA1 significantly (*p* < 0.05) upregulated the expression of miR-125b-5p and miR-451a relative to anti-CD3/CD28, while miR-18a-5p, miR-17-5p, miR-20a-5p and miR-135b-5p were downregulated. Similar to Fig. 3 multiple other miRNA were also differentially expressed between IA1 and anti-CD3/CD28 activation though they did not reach significance in the volcano plot analyses (though if compared to resting PBMC they are different). Interestingly, the miRNA expression profiles between IA1 and TA1 were not statistically significantly different (Fig. 4B), though, as also seen in Fig. 3, miR-298, miR-214-3p, miR-302a-3p and miR-206 were over-expressed in IA1 relative to TA1. Finally, the expression of miR-149-5p and miR-18b-5p were significantly (*p* < 0.05) upregulated in PBMC treated with IA1 when compared to the same donor PBMC treated with IA2 (Fig. 4C).

**Fig. 5:**
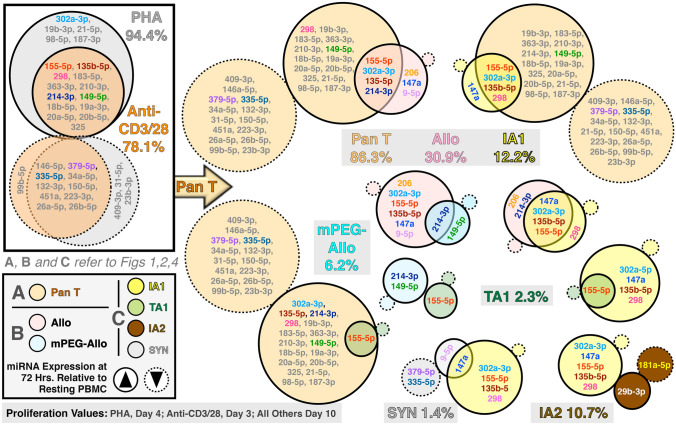
Venn diagram comparison between Pan T Cell (**A**), Allo (**B**) and Secretome (**C**) activation of resting human PBMC. Over-expressed miRNA are listed in solid line bubbles, while under-expressed miRNA are in dashed line bubbles. Overlapped areas indicate miRNA with similarly increased/decreased miRNA expression. The bubble color indicates different activation treatments. miRNA of interest (relative to Figs. 3, 4, 5; Supplementary Table 1S) are in color. As shown, pan T cell, allo- and secretome activation exerted distinct patterns of miRNA expression in treated resting PBMC and their putative functions are described in Supplementary Table 2S. As shown, Pan T cell activators exhibited huge changes (up/down) in a large number of miRNA. In contrast, alloactivation (both the control- and mPEG-MLR) showed dramatically reduced miRNA changes and the secretome products showed an even further reduction in the differentially expressed miRNA. Proliferation values for each approach/agent are provided within the figure

In sum, previous clustergram (Yang et al. 2019), and our current studies utilizing log_2_ fold change and volcano plot analyses demonstrate the differential activation strategies yielded dramatically different miRNA expression profiles that in turn resulted in significant differences in T-cell activation and subset differentiation. To better understand these differences, an integrative Venn diagram analysis was done using all three sets of data (Fig. 5) to differentially compare the Pan T-cell, allorecognition and secretome activation. As demonstrated, Pan T-cell activation using PHA and anti-CD3/CD28 yielded similar, though not identical, changes in miRNA expression (solid circles = over expression; dashed circles = reduced expression; overlap are miRNA in common). For further comparison purposes, we averaged the miRNA expression profile and proliferation rates of PHA and anti-CD3/CD28 to represent the efficacy of Pan T-cell activation strategy. In contrast to Pan T-cell activation, the miRNA changes induced by allorecognition were much more discreet (relative to resting PBMC) and highly limited when compared to the Pan T-cell activators. Moreover, allorecognition resulted in a significant reduction in cell proliferation (pan T: 86.3% versus 30.9% for allorecognition). Similar to the allorecognition response, the allo-derived IA1 secretome also induced a limited miRNA response pattern relative to Pan T-cell activation and, not surprisingly, similar to the pattern of expression observed in the alloresponse but with the increased expression of miR-298 and decreased expression of miR-206 and miR-214-3p. While some miRNA are in common to the pan T cell, Allo, IA1 and IA2 pro-inflammatory responses (overlaps in Venn diagrams), some of these (e.g., miR-155-5p) are also implicated in the tolerogenic TA1 and mPEG-Allo responses. This again argues that the “pattern of miRNA expression” (Fig. 3B) encompassing increases, decreases and static levels of multiple, rather than a specific (single or small number), of miRNA is crucial.

Importantly, the biomanufaturing process is of importance. This is most obvious in comparing the MLR vs mPEG-MLR and IA1 vs IA2 miRNA patterns. While IA1 and IA2 stimulated similar proliferative effects (12.2 ± 1.2 and 10.7 ± 0.5%, respectively), their impacts on CD4/CD8 differentiation (Fig. 2) were vastly different and, as observed in our previous study, IA1 and IA2 exhibited distinct biological activities and anti-cancer mechanisms (Scott et al. 2019; Wang et al. 2015; Yang et al. 2019). Indeed, as shown in Fig. 5, PBMC pre-treated with IA1 and IA2 induced entirely different miRNA expression that resulted in vastly different responses to cancer cells. IA2 but not IA1, increased the expression of cellular miR-29b-3p which has been shown to promote cellular apoptosis in cancers (Li et al. 2013; Zeng et al. 2019). Consistently, miR-181a-5p, an oncogene in various tumors suppressing apoptosis, was downregulated by IA2, further supporting our previous findings that IA2 inhibited cancer cell proliferation via an apoptosis-associated mechanism (Lai et al. 2018a,b; Yang et al. 2018).

In conclusion, these studies demonstrate that Pan T-cell activators, alloactivation and secretome-based therapeutics induced differential patterns of miRNA expression in leukocytes, which governs/reflects significant differences in cell proliferation, differentiation and immunological activity. Pan T-cell activators induced massive miRNA alteration profiles and T-cell proliferation relative to resting cells. Allo-MLR demonstrated a more discriminatory alteration of miRNA expression relative to Pan T-cell activators, while mPEG-MLR diminished allorecognition related miRNA expression. Importantly, the magnitude of changes and pattern of miRNA expression in the IA1 secretome was similar to that observed with a control MLR and exerted a pro-inflammatory effect similar to the MLR response (Fig. 3). In contrast, resting cell generated secretome SYN had minimal effects on recipient resting PBMC. Of interest, the HeLa-MLR derived IA2 therapeutic exhibited distinct alterations to the leukocyte miRNA expression profile, suggesting an apoptosis-associated immunomodulatory and anti-cancer pathway.

## Discussion

Highly controlled systemic and, in some cases, localized immunomodulation of the T cell-mediated immune response has the potential to be a potent weapon in the treatment of both autoimmune diseases and cancers. Biologically, T-cell-mediated immune responses encompass a broad range of subpopulations that give rise to a “net” inflammatory or tolerogenic environment. Importantly, both the inflammatory and tolerogenic responses must be highly regulated to prevent undesirable immunological consequences. From a clinical perspective, the regulation of the inflammatory-tolerance continuum has typically focused the induction/administration of agents that result in the differential production of effector and/or Treg cells. While multiple drugs (e.g.*,* cyclosporine, PHA) and biologics (e.g.*,* etanercept and anti-CD3) have been explored and, in some case used successfully in the clinic, these agents do not give rise to a well-regulated immune response; thus, placing the patients at risk (Barrett 1997; Barrett and Childs 2000; Deng et al. 2001; Fabre 2001; Fathman and Myers 1992; Feutren 1992; Khanna et al. 2003; Liao et al. 2011; Mire-Sluis et al. 1987; Scott 2014; Starnes 1992a,b; Stathopoulos et al. 2008; Trickett and Kwan 2003).

To replicate the normally highly regulated control of the in vivo inflammatory-tolerance continuum, our laboratory has demonstrated that polymer-based bioengineering of immune cells can be used either directly, or more practically, via biomanufactured secretome miRNA-based therapeutics, to systemically modify the immune response (Kang et al. 2017; Kyluik-Price et al. 2014; Kyluik-Price and Scott 2016; Le and Scott 2010; Le et al. 2012; Murad et al. 1999; Scott et al. 1997; Wang et al. 2011, 2012, 2015; Yang et al. 2019). As shown in these studies, miRNA secretome therapeutics, results in the differential production of T-cell subpopulations necessary to discreetly produce well-controlled tolerogenic or pro-inflammatory responses both in vitro and in vivo (Scott et al. 2019; Wang et al. 2011, 2015; Yang et al. 2019). Indeed, as shown in vivo, the tolerogenic TA1 secretome systemically increased Treg cells while downregulating pro-inflammatory T-cell subsets in the NOD model of Type 1 diabetes; thus, inhibiting islet cell destruction and the development of overt diabetes (Wang et al. 2015). Conversely, treatment of naïve human PBMC (or murine splenocytes) with the IA1 pro-inflammatory secretome enhanced the pro-inflammatory T-cell proliferation resulting in the increased in vitro killing of tumor cells (Yang et al. 2019).

Mechanistically, as shown in this study, one can correlate the proliferation and subset differentiation of T cells (Fig. 2) with the differential miRNA profiles (Figs. 3, 4, 5) of broad acting agonists (e.g.*,* PHA/PMA) relative to the increasingly controlled (allorecognition > IA1 > TA1) immunomodulatory approaches. Indeed, while Pan T-cell activators induced runaway proliferation and massive miRNA changes, the differential miRNA expression profile arising from the allorecognition-based IA1 secretome therapeutic induced a highly controlled pro-inflammatory response relative to both the Pan T-cell activators and allorecognition itself (Figs. 2, 5). The restrained, yet biologically effective (Yang et al. 2019), inflammatory response induced by IA1 is important as stronger pro-inflammatory approaches (e.g., mitogen and even allorecognition) can generate overly robust responses leading to bystander cell injury and, in worst case scenarios, cytokine release syndrome (Kang et al. 2017; Kyluik-Price et al. 2014; Kyluik-Price and Scott 2016; Murad et al. 1999; Scott et al. 2019; Wang et al. 2011, 2015; Yang et al. 2019). In contrast, PEGylated-PBMC and TA1, both of which induced a systemic and persistent tolerogenic state, exhibited significantly different miRNA profiles (Kang et al. 2017; Kyluik-Price et al. 2014; Kyluik-Price and Scott 2016; Murad et al. 1999; Scott et al. 2019; Wang et al. 2011, 2015; Yang et al. 2019). In aggregate, the results shown here and in our previous publications suggest that secretome-based therapeutics have the potential to be potent, but controllable, tools for systemic immunomodulation.

Hence, while clinical immunologists have focused on cytokine/chemokine immunotherapy, miRNA-based biotherapeutics may prove to be biologically more useful in that, with appropriate formulations, the strength and type of the immune response may be more titratable. miRNA have been increasingly found to be key bioregulatory messengers as both external (i.e., extracellular miRNA) as well intracellular effectors of gene activation and mRNA expression. Indeed, the importance of miRNA in immune cell proliferation and differentiation has been extensively studied (Carissimi et al. 2014; Chen et al. 2013; Dudda et al. 2013; Li et al. 2007; Lu et al. 2009; Rossi et al. 2011; Rusca et al. 2012; Shin et al. 2013; Teteloshvili et al. 2015; Wu et al. 2007; Xiao et al. 2007). Moreover, the utility of miRNA in cancer diagnosis (i.e., biomarkers) and treatment (small interfering RNA) has gained increasing attention during the past decade (Bovy et al. 2015; Chen et al. 2008, 2012; Chim et al. 2008; Hayes et al. 2014; Kosaka et al. (2010); Lawrie et al. 2008; Mitchell et al. 2008; Munich et al. 2012; Okoye et al. 2014; Park et al. 2009; Turchinovich et al. 2012; Weber et al. 2010). As documented in this study, pan T cell, allorecognition and secretome activation of resting PBMC induce dramatically different miRNA expression patterns within the treated PBMC (Figs. 3, 4, 5) that lead to different proliferation and differentiation responses.

**Fig. 6:**
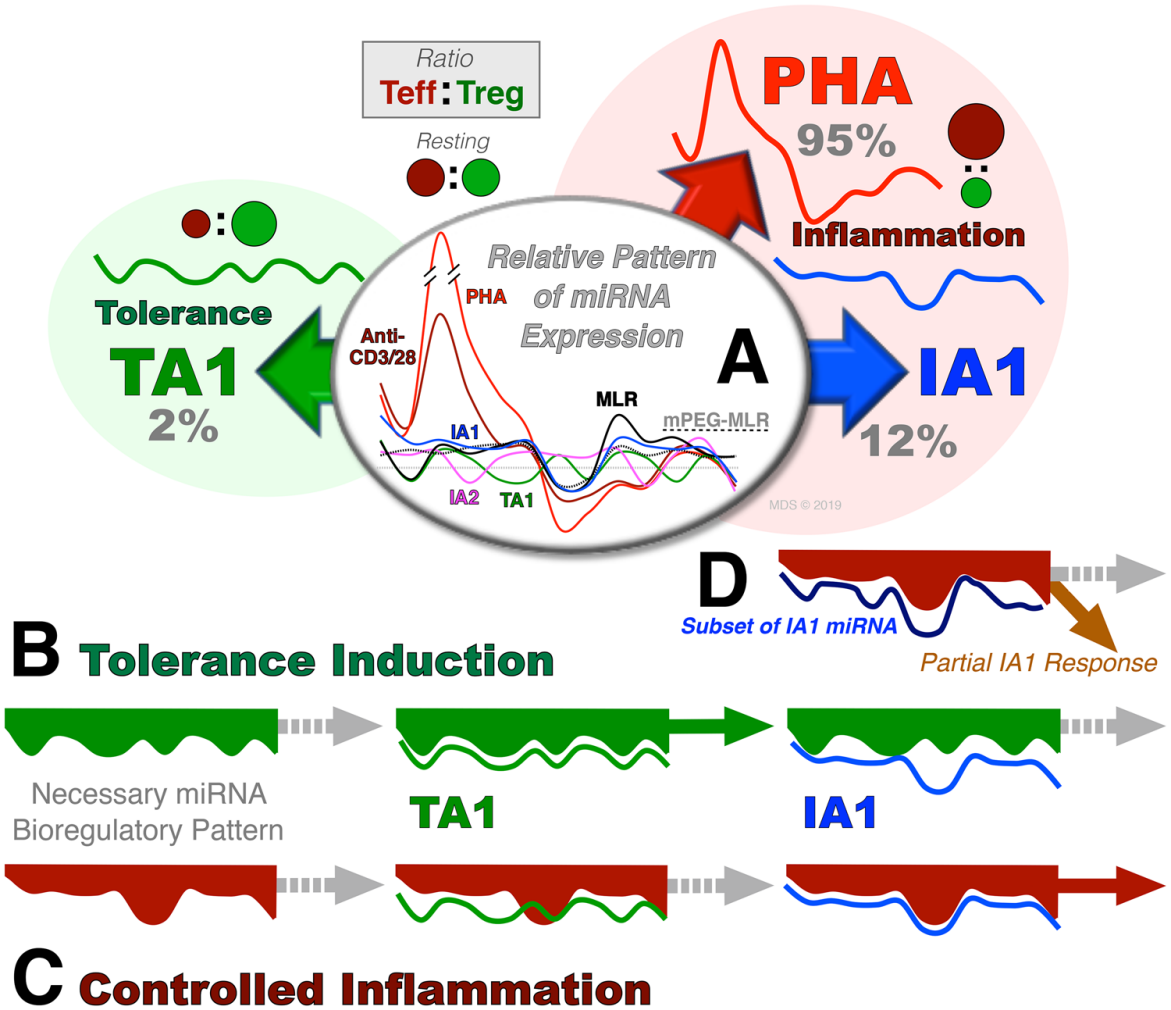
Bioregulation occurs consequent to pattern specificity of miRNA expression**.** miRNA are “low fidelity” due to the promiscuous nature of miRNA: a single miRNA can interact with hundreds of genes and a single gene can interact with multiple miRNA. Hence, the “Pattern of miRNA” (both species and relative abundance) governs the biologic response in a “lock and key” manner. **A** PBMC activation with pan T cell, allorecognition-driven, and secretome products yield dramatically different miRNA expression patterns and proliferation rates. **B**–**C** Using a “lock and key” analogy, TA1 (**B**) and IA1 (**C**) induce miRNA expression profiles that *exclusively* produce either a tolerogenic (TA1) or pro-inflammatory (IA1) effect on treated PBMC. **D** However, if partial pattern parity exists, aspects of the biological response may be retained. As shown, a similar IA1-like pattern may induce a partial response. Indeed, per Fig. 6, IA1 may be viewed as a subset of the alloresponse which itself is a subset of Pan T-cell activators. The disparity in the miRNA expression between these three pro-inflammatory responses is similarly reflected in the induction of proliferation noted between the activators

Typically, due to the inherent reductionist nature of science, large increases or decreases of a single miRNA have been focused on as being responsible for the observed changes in proliferation and differentiation in both autoimmune diseases and pro-inflammatory states. However, our understanding of miRNA-mediated bioregulation is still in its infancy. In light of the fact that a single miRNA can interact with hundreds of genes, and a single gene can interact with tens to hundreds of miRNA, this “low fidelity” is suggestive that “distinct patterns of miRNA expression” rather than changes in a single miRNA are key to inducing complex (e.g., tolerance or controlled inflammation) bioregulatory changes (Chen et al. 2013; Mavrakis et al. 2011; Scott et al. 2019; Wang et al. 2015; Yang et al. 2019). Indeed, examining the miRNA expression profiles (Figs. 3, 4, 5) induced by Pan T-cell activators (PHA or anti-CD3-CD28), allorecognition, or our secretome-derived TA1 and IA1 products shows significant variations in both cell phenotypes and the miRNA expression profiles. Singling out a single miRNA is difficult to do. For example, miR-155-5p is a crucial component of both tolerogenic and pro-inflammatory responses, but other supporting miRNA differ (e.g.*,* Fig. 5). However, if one appreciates the complexity of miRNA, one can begin to look at “patterns of expression” as governing the bioregulatory response. Schematically this bioregulatory process is described in Fig. 6A in which the unique pattern of miRNA expression observed in Fig. 3B is presented and governs the biologic response in a “lock and key” manner. Hence, the “pattern of expression” of TA1 replicates the expression profile to induce a tolerogenic response (i.e., increased Treg, decreased Teff (Kang et al. 2017; Scott et al. 2019; Wang et al. 2015; Yang et al. 2019) Fig. 6B); while the IA1 expression profile cannot induce tolerance due to its “pattern disparity”. In contrast, IA1 effectively unlocks a controlled inflammatory response (Fig. 6C) that is in stark contrast to the pan T cell or even alloresponse-induced inflammation which are characterized by significantly more expansive miRNA expression profiles (Figs. 3, 4, 5). Moreover, it is possible to also hypothesize that partial pattern homology could induce a partial response (Fig. 6D); indeed, while TA1 is shown as the example, IA1 is a clear subset of the pan T cell and Alloresponse miRNA response patterns (Fig. 5). The complexity of this “lock and key” regulatory mechanism readily explains the often highly disparate functions assigned to a single miRNA in the literature (see Supplementary Tables 1S and 2S). Hence, despite the reductionist nature of science, understanding miRNA bioregulation may need to become more focused on the overall expression patterns including not just large changes (up/down), but more modest increases or decreases, and even miRNA that remain static responses during complex biological responses. By mimicking the “pattern of miRNA expression”, it may be possible to produce secretome, or purified miRNA, products that can replicate the desired biological response. Indeed, the TA1 therapeutic induced a systemic in vivo tolerance resulting in disease attenuation in the NOD mouse model of Type 1 diabetes (Wang et al. 2015). In contrast, treatment of resting PBMC with the IA1 therapeutic significantly enhanced their anti-cancer efficacy in vitro (Yang et al. 2019). Of note, secretome-derived miRNA-based therapeutics could be used either independently or in conjunction with other cell-based therapies (e.g., CAR T cells).

In light of our proposed model of miRNA regulation, a question that clearly arises is whether the “complexity” of the described miRNA secretome therapeutics pose a potential problem for drug development. While clearly some reductionism in the formulary is necessary, it is important to remember that miRNA bioregulation is “messy” due to its low fidelity, highly promiscuous, nature. As we have argued, to replicate normal bioregulation, the miRNA mixture likely needs to contain a complex array of miRNA consisting of miRNA species of increasing, decreasing and static abundance. Hence, miRNA therapeutics would partially replicate the complexity of clinically used intravenous immune globulin which contains purified IgG from thousands of individuals. Moreover, further development of secretome-based drugs would benefit from a more detailed analysis of the effects on the miRNA therapeutics on T cells subpopulations over that shown in our previous publications (Kang et al. 2017; Kyluik-Price et al. 2014; Kyluik-Price and Scott 2016; Le and Scott 2010; Murad et al. 1999; Scott et al. 2019; Wang et al. 2011, 2015; Yang et al. 2019). These studies would be informative and could be assessed by single cell analysis of naïve lymphocyte using progressively reduced sets of miRNA. However, these studies would need to be tempered by the knowledge that they would, at most represent only a small, potentially non-representative, subset of the total T-cell population necessary for inducing a systemic effect. Indeed, the “yin-yang” of competing subsets of the global immune response is a crucial component of “biological control”. Hence, the most appropriate model for testing the biological efficacy of the miRNA-based secretome therapeutics remains the functional “net” effects on the immune state of complex cell populations and intact animals.

Finally, an important finding of this study to note is that Pan T-cell activation bears little resemblance to allorecognition with regards to the miRNA induced, which, in turn, will govern the regulation, differentiation and proliferation of resting T cells. While many publications have utilized Pan T-cell activators (e.g., PHA or anti-CD3/anti-CD28) to model T-cell-mediated pathologies (e.g., allorecognition, transplant rejection, GvHD, autoimmunity and inflammation) (Maciel et al. 1976; Trickett and Kwan 2003), our results clearly indicate that there are very significant differences in the miRNA response pattern of human PBMC at 72 h (well into the proliferation and differentiation cycle) between the activation strategies (Fig. 5). Indeed, Pan T-cell stimulators (~ 90% activation) induce massive miRNA alterations (Figs. 3, 4, 5), that yield non-specific overactivation and significant bystander injuries (Han and Takita 1972; Maciel et al. 1976; Suntharalingam et al. 2006). In contrast, allorecognition generates a more discriminatory T-cell response (proliferation/differentiation and miRNA expression; Figs. 3, 4, 5) as only 1–10% of an individual’s T lymphocytes are alloreactive (Abul et al. 2015; Nisbet et al. 1969). Despite the “low” number of potentially reactive cells, the alloresponse is still quite potent as exemplified by the severity of GvHD (Nowak 2008). Not surprisingly, the alloresponse has been studied in the context of cancer immunity for decades (Abul et al. 2015; de Gruijl et al. 2008; Gong et al. 2000; Kugler et al. 2000; Stathopoulos et al. 2008). Although mitogens and anti-CD3/CD28 have been widely used to activate and expand immune cells ex vivo in cancer immunotherapies, our findings suggest that Pan T-cell activators do not model normal allorecognition-based stimulation and a reliance of these agents may lead to artifactual conclusion regarding immune responses, and could underlie the cytokine release syndrome commonly seen in overly robust T-cell responses to “non-self” (Han and Takita 1972; Maciel et al. 1976; Suntharalingam et al. 2006).

In summary, T-cell activation is a crucial element of the immune response. Recent studies have shown that miRNAs play a key role in tuning the immune response toward either an inflammatory or tolerogenic pathway. However, as demonstrated here, activation stimuli show significant variations in their generation of miRNA and, hence, the induced immunological response (strength, T-cell differentiation, inflammation, tolerance, etc.). Indeed, our findings highlight the significant disparity between mitogen and alloactivation-induced proliferation suggesting that mitogen stimulation is a poor model of “normal” T-cell activation and may underlie their adverse effects when used clinically. In the development of miRNA-based therapeutics, we propose that the “pattern of miRNA production”, rather than a single miRNA, is of paramount importance as a single miRNA is “low fidelity” in that it can interact with 10–100’s of genes. Thus, we propose that miRNA-based bioregulation occurs via a “lock and key” mechanism based on the “pattern of miRNA expression” of a number of miRNA including increased, decreased and unchanged miRNA. In light of this observation, the development of miRNA-based therapeutics may be best achieved, NOT by focusing on a single miRNA, but rather via a cocktail of miRNA that mimic (at least partially) the naturally occurring miRNA expression profiles leading to controlled inflammation (e.g., IA1) or tolerance induction (e.g., TA1). To this end, the biomanufacturing of immunomodulatory, miRNA-enriched, secretome biotherapeutics may provide potent tools for the systemic treatment of both autoimmune diseases (TA1) and cancer (IA1) (Scott et al. 2019; Wang et al. 2015; Yang et al. 2019). The successful development of secretome-derived, miRNA therapeutics that replicate specific bioregulatory events may prove useful in the treatment of autoimmune diseases or enhancing the endogenous immune response to cancer while reducing the potential adverse risks of more non-specific immunomodulatory approaches.

## Supplementary Information

Below is the link to the electronic supplementary material.

Supplementary file1 (DOCX 26 KB)

Supplementary file2 (DOCX 60 KB)

## Data Availability

All data analyzed in this study are included within the included figures and tables, Supplemental File 1, or are available from the authors upon reasonable request.

## Abbreviations

miRNA: MicroRNA
PBMC: Peripheral blood mononuclear cells
Treg: Regulatory T cells
Teff: T effector cell encompassing subsets such as Th17 and Th1
mPEG: Methoxypolyethylene glycol
PHA: Phytohemagglutinin
MLR: Mixed lymphocyte reaction
HeLa: Epithelial cancer cell line
SYN: Control secretome product derived from resting PBMC
TA1: Tolerance agent 1; derived from mPEG-MLR
IA1: Inflammatory agent 1; derived from control MLR
IA2: Inflammatory agent 2; derived from PBMC-HeLa cell co-culture
