# Supplementary material for: Differential Leukocyte MicroRNA Responses Following Pan T Cell, Allorecognition and Allosecretome-Based Therapeutic Activation
Source: Arch Immunol Ther Exp (Warsz). 2021 Oct 22;69(1):30. doi: 10.1007/s00005-021-00634-5 (PMC8536625; doi:10.1007/s00005-021-00634-5)
Supplement: Supplementary file 1 — Supplementary file1 (DOCX 26 KB) [file 5_2021_634_MOESM1_ESM.docx]

**Table 1S. Putative Functions of 13 Selected Key miRNA shown in Figure 3.**

| **hsa-miR-** | **Key Activator(s)** | **Putative Function(s)** |
| --- | --- | --- |
| **155-5p** | ↑ in Pan T, IA1, TA1 | miR-155 expression inhibits malignant growth *in vivo*.[1] *see also* miR-147a. |
| **298** | ↑ in Pan T, IA1 | Implicated (along with miR-296-3p) in increased resistance of mammalian pancreatic α cells to cytokine-induced apoptosis.[2] |
| **135b-5p** | ↑ in Pan T, IA1 | Reported to be an oncogenic miRNA enhancing cancer cell invasive and metastasis *in vivo*.[3] Important in the regulation of inflammation. miR-135b expression in inflammation is regulated by IL-1R1 in a regulatory feedback mechanism to resolve inflammation.[4] |
| **149-5p** | ↑ in Pan T | Expression is inversely associated with inflammation and has regulatory effects on TNF-α, IL-1ß and IL-6 and is involved in the regulation of apoptosis.[5,6] |
| **214-3p** | ↑ in Pan T | Predicted to target a number of genes regulating apoptosis, immune cell proliferation and angiogenesis. Upregulation in Hela cells reduced cell growth. In contrast, elevated expression has also been shown to inhibit chemotherapy effectiveness.[7–9] |
| **302a-3p** | ↑ in PHA, IA1 | Shown to be able to reprogram human cancer cells to human embryonic stem cell (hESC)–like pluripotent cells with a slow cell cycle rate and dormant cell-like morphology.[10,11] Inhibits CDK2 and CDK4/6 cell cycle pathways.[12] |
| **335-5p** | ↓ in Pan T | Has been identified as a cancer suppressor and found to be downregulated in gastric cancer and involved in tumorigenesis.[13–15]Overexpression of miR-335-5p promotes osteogenic differentiation[13] and bone formation and regeneration in mice.[16] |
| **379-5p** | ↓ in Pan T | Is reported to be a tumor suppressor involved in multiple cancers, especially in glioblastoma multiforme.[17] Reported to (with miR-374-5p and miR-503-5p) regulate proliferation and hypertrophic differentiation of growth plate chondrocytes in male rats.[18] |
| **147a** | ↑ IA1 | Implicated along with miR-155 (*see this table*) and miR-21 (*see* **additional file 1**) in regulation of Toll-like receptors (TLRs). The dysregulation of these miRNAs may be involved in inflammatory diseases and cancers.[19] |
| **9-5p** | ↑ IA1 | Induced by TLR4 activation as well as TLR2 and TLR7/8 agonists and by the proinflammatory cytokines (TNF-α and IL-1β, but not by IFN-γ). [20] However, other studies have shown that miR-9 expression is reduced in some cancers.[21] |
| **206** | ↓ in TA1 versus IA1 | Inverse relationship between miR-206 expression and Th17 cells in an inflammatory disease (dermatomyositis).[22] |
| **29b-3p** | ↑ in IA2 | miR-29 family has diverse roles in cancer. Can function as a tumor suppressor, attenuating cancer progression via promoting tumor cell apoptosis and by reducing cell proliferation. However, as a tumor promoter, miR-29 mediates epithelial-mesenchymal transition (EMT) and promotes metastasis in breast cancer and colon cancer.[23] |
| **181a-5p** | ↓ in IA2 | Promotes proliferation and invasion and inhibits apoptosis of cervical cancer cells.[24] Expression is upregulated in gastric cancer and correlated with invasion and facilitates cell proliferation in acute lymphoblastic leukemia.[25,26] In contrast, other studies report that it inhibits cancer cell proliferation, migration, invasion and angiogenesis via downregulation of matrix metalloproteinase-14 (MMP-14) and/or in targeting Kras.[27,28]. |

**REFERENCES**

1. Babar IA, Cheng CJ, Booth CJ, Liang X, Weidhaas JB, Saltzman WM, Slack FJ. Nanoparticle-based therapy in an in vivo microRNA-155 (miR-155)-dependent mouse model of lymphoma. Proc Natl Acad Sci U S A. 2012;109:E1695-704. 10.1073/pnas.1201516109

2. Barbagallo D, Piro S, Condorelli AG, Mascali LG, Urbano F, Parrinello N, Monello A, Statello L, Ragusa M, Rabuazzo AM, Di Pietro C, Purrello F, Purrello M. miR-296-3p, miR-298-5p and their downstream networks are causally involved in the higher resistance of mammalian pancreatic α cells to cytokine-induced apoptosis as compared to β cells. BMC Genomics. 2013;14:62. 10.1186/1471-2164-14-62

3. Lin CW, Chang YL, Chang YC, Lin JC, Chen CC, Pan SH, Wu CT, Chen HY, Yang SC, Hong TM, Yang PC. MicroRNA-135b promotes lung cancer metastasis by regulating multiple targets in the Hippo pathway and LZTS1. Nat Commun. 2013;4:1877. 10.1038/ncomms2876

4. Halappanavar S, Nikota J, Wu D, Williams A, Yauk CL, Stampfli M. IL-1 receptor regulates microRNA-135b expression in a negative feedback mechanism during cigarette smoke-induced inflammation. J Immunol. 2013;190:3679-3686. 10.4049/jimmunol.1202456

5. Lin RJ, Lin YC, Yu AL. miR-149* induces apoptosis by inhibiting Akt1 and E2F1 in human cancer cells. Mol Carcinog. 2010;49:719-727. 10.1002/mc.20647

6. Santini P, Politi L, Vedova PD, Scandurra R, Scotto d’Abusco A. The inflammatory circuitry of miR-149 as a pathological mechanism in osteoarthritis. Rheumatol Int. 2014;34:711-716. 10.1007/s00296-013-2754-8

7. Bar-Eli M. Searching for the ‘melano-miRs’: miR-214 drives melanoma metastasis. EMBO J. 2011;30:1880-1881. 10.1038/emboj.2011.132

8. Yang Z, Chen S, Luan X, Li Y, Liu M, Li X, Liu T, Tang H. MicroRNA-214 is aberrantly expressed in cervical cancers and inhibits the growth of HeLa cells. IUBMB Life. 2009;61:1075-1082. 10.1002/iub.252

9. Zhang XJ, Ye H, Zeng CW, He B, Zhang H, Chen YQ. Dysregulation of miR-15a and miR-214 in human pancreatic cancer. J Hematol Oncol. 2010;3:46. 10.1186/1756-8722-3-46

10. Lin SL, Chang DC, Chang-Lin S, Lin CH, Wu DT, Chen DT, Ying SY. Mir-302 reprograms human skin cancer cells into a pluripotent ES-cell-like state. RNA. 2008;14:2115-2124. 10.1261/rna.1162708

11. Barroso-del Jesus A, Lucena-Aguilar G, Menendez P. The miR-302-367 cluster as a potential stemness regulator in ESCs. Cell Cycle. 2009;8:394-398. 10.4161/cc.8.3.7554

12. Lin SL, Chang DC, Ying SY, Leu D, Wu DT. MicroRNA miR-302 inhibits the tumorigenecity of human pluripotent stem cells by coordinate suppression of the CDK2 and CDK4/6 cell cycle pathways. Cancer Res. 2010;70:9473-9482. 10.1158/0008-5472.CAN-10-2746

13. Zhang J, Tu Q, Bonewald LF, He X, Stein G, Lian J, Chen J. Effects of miR-335-5p in modulating osteogenic differentiation by specifically downregulating Wnt antagonist DKK1. J Bone Miner Res. 2011;26:1953-1963. 10.1002/jbmr.377

14. Li H, Xie S, Liu M, Chen Z, Liu X, Wang L, Li D, Zhou Y. The clinical significance of downregulation of mir-124-3p, mir-146a-5p, mir-155-5p and mir-335-5p in gastric cancer tumorigenesis. Int J Oncol. 2014;45:197-208. 10.3892/ijo.2014.2415

15. Sandoval-Bórquez A, Polakovicova I, Carrasco-Véliz N, Lobos-González L, Riquelme I, Carrasco-Avino G, Bizama C, Norero E, Owen GI, Roa JC, Corvalán AH. MicroRNA-335-5p is a potential suppressor of metastasis and invasion in gastric cancer. Clin Epigenetics. 2017;9:114. 10.1186/s13148-017-0413-8

16. Zhang L, Tang Y, Zhu X, Tu T, Sui L, Han Q, Yu L, Meng S, Zheng L, Valverde P, Tang J, Murray D, Zhou X, Drissi H, Dard MM, Tu Q, Chen J. Overexpression of MiR-335-5p Promotes Bone Formation and Regeneration in Mice. J Bone Miner Res. 2017;32:2466-2475. 10.1002/jbmr.3230

17. Laddha SV, Nayak S, Paul D, Reddy R, Sharma C, Jha P, Hariharan M, Agrawal A, Chowdhury S, Sarkar C, Mukhopadhyay A. Genome-wide analysis reveals downregulation of miR-379/miR-656 cluster in human cancers. Biol Direct. 2013;8:10. 10.1186/1745-6150-8-10

18. Jee YH, Wang J, Yue S, Jennings M, Clokie SJ, Nilsson O, Lui JC, Baron J. mir-374-5p, mir-379-5p, and mir-503-5p Regulate Proliferation and Hypertrophic Differentiation of Growth Plate Chondrocytes in Male Rats. Endocrinology. 2018;159:1469-1478. 10.1210/en.2017-00780

19. Quinn SR, O’Neill LA. A trio of microRNAs that control Toll-like receptor signalling. Int Immunol. 2011;23:421-425. 10.1093/intimm/dxr034

20. Bazzoni F, Rossato M, Fabbri M, Gaudiosi D, Mirolo M, Mori L, Tamassia N, Mantovani A, Cassatella MA, Locati M. Induction and regulatory function of miR-9 in human monocytes and neutrophils exposed to proinflammatory signals. Proc Natl Acad Sci U S A. 2009;106:5282-5287. 10.1073/pnas.0810909106

21. Lehmann U, Hasemeier B, Christgen M, Müller M, Römermann D, Länger F, Kreipe H. Epigenetic inactivation of microRNA gene hsa-mir-9-1 in human breast cancer. J Pathol. 2008;214:17-24. 10.1002/path.2251

22. Tang X, Tian X, Zhang Y, Wu W, Tian J, Rui K, Tong J, Lu L, Xu H, Wang S. Correlation between the frequency of Th17 cell and the expression of microRNA-206 in patients with dermatomyositis. Clin Dev Immunol. 2013;2013:345347. 10.1155/2013/345347

23. Jiang H, Zhang G, Wu JH, Jiang CP. Diverse roles of miR-29 in cancer (review). Oncol Rep. 2014;31:1509-1516. 10.3892/or.2014.3036

24. Yang M, Zhai X, Ge T, Yang C, Lou G. miR-181a-5p Promotes Proliferation and Invasion and Inhibits Apoptosis of Cervical Cancer Cells via Regulating Inositol Polyphosphate-5-Phosphatase A (INPP5A). Oncol Res. 2018;26:703-712. 10.3727/096504017X14982569377511

25. Chen G, Shen ZL, Wang L, Lv CY, Huang XE, Zhou RP. Hsa-miR-181a-5p expression and effects on cell proliferation in gastric cancer. Asian Pac J Cancer Prev. 2013;14:3871-3875. 10.7314/APJCP.2013.14.6.3871

26. Lyu X, Li J, Yun X, Huang R, Deng X, Wang Y, Chen Y, Xiao G. miR-181a-5p, an inducer of Wnt-signaling, facilitates cell proliferation in acute lymphoblastic leukemia. Oncol Rep. 2017;37:1469-1476. 10.3892/or.2017.5425

27. Li Y, Kuscu C, Banach A, Zhang Q, Pulkoski-Gross A, Kim D, Liu J, Roth E, Li E, Shroyer KR, Denoya PI, Zhu X, Chen L, Cao J. miR-181a-5p Inhibits Cancer Cell Migration and Angiogenesis via Downregulation of Matrix Metalloproteinase-14. Cancer Res. 2015;75:2674-2685. 10.1158/0008-5472.CAN-14-2875

28. Ma Z, Qiu X, Wang D, Li Y, Zhang B, Yuan T, Wei J, Zhao B, Zhao X, Lou J, Jin Y, Jin Y. MiR-181a-5p inhibits cell proliferation and migration by targeting Kras in non-small cell lung cancer A549 cells. Acta Biochim Biophys Sin (Shanghai). 2015;47:630-638. 10.1093/abbs/gmv054
