# Supplementary material for: Differential Leukocyte MicroRNA Responses Following Pan T Cell, Allorecognition and Allosecretome-Based Therapeutic Activation
Source: Arch Immunol Ther Exp (Warsz). 2021 Oct 22;69(1):30. doi: 10.1007/s00005-021-00634-5 (PMC8536625; doi:10.1007/s00005-021-00634-5)
Supplement: Supplementary file 2 — Supplementary file2 (DOCX 60 KB) [file 5_2021_634_MOESM2_ESM.docx]

**Table 2S.**  Differential effects of Pan T cell (A), Allo (B) and Secretome-based activation (C) of human PBMC on (hsa-) miRNA expression levels relative to Resting (Fresh) PBMC. miRNA levels were assessed 72 hours post treatment using 3 strategies: volcano plot (v), clustergram (c) and net 2-fold change (2f) analyses. Changes in miRNA expression are denoted as follows: **I** = Increased; **D** = Decreased; and **-** = Unchanged. CD: anti-CD3/CD28. MLR: mixed lymphocyte reactions.

|  | A – Pan T Cell Activation | | | | | | B – Alloactivated PBMC | | | | | | C – Secretome Product Activation | | | | | | | | | | | |  |
| --- | --- | --- | --- | --- | --- | --- | --- | --- | --- | --- | --- | --- | --- | --- | --- | --- | --- | --- | --- | --- | --- | --- | --- | --- | --- |
|  | **CD** | | | **PHA** | | | **mPEG-MLR** | | | **Control-MLR** | | | **SYN** | | | **TA1** | | | **IA1** | | | **IA2** | | | **Putative Function(s)** |
| **hsa-miR-xxx** | v | c | 2f | v | c | 2f | v | c | 2f | v | c | 2f | v | c | 2f | v | c | 2f | v | c | 2f | v | c | 2f |  |
| **203a-3p** | - | - | - | - | - | - | - | D | - | - | I | - | - | - | - | - | - | - | - | - | - | - | I | - | miR-203 has been found overexpressed in pancreatic adenocarcinoma and is correlated with poor prognosis. The expression of this miR-203 is induced by various cytokines, including IL6 and IFN-γ. [1] |
| **206** | - | I | - | - | - | - | - | I | - | I | I | I | - | I | - | - | - | - | - | I | - | - | - | - | Inverse realtionship beetween miR-206 expression and Th17 cells in an inflammatory disease (dermatomyositis). [2] |
| **302a-3p** | - | - | - | I | I | I | - | I | - | - | I | I | - | - | - | - | - | - | I | I | I | - | - | - | Studies have shown that miR-302 is able to reprogram human cancer cells to human embryonic stem cell (hESC)–like pluripotent cells with a slow cell cycle rate and dormant cell-like morphology. [3,4] Inhibits CDK2 and CDK4/6 cell cycle pathways. [5] |
| **29b-3p** | - | - | - | - | - | - | - | I | - | - | I | - | - | - | - | - | - | - | - | I | - | I | I | I | miR-29 family has diverse roles in cancer. As a tumor suppressor, miR-29 restrains cancer progression by promoting tumor cell apoptosis, by suppressing DNA methylation of tumor-suppressor genes, by reducing proliferation of tumors and by increasing chemosensitivity. However, as a tumor promoter, miR-29 mediates epithelial-mesenchymal transition (EMT) and promotes metastasis in breast cancer and colon cancer. [6] |
| **147a** | - | - | - | - | - | - | - | - | I | I | I | I | I | I | I | - | - | - | I | I | I | - | - | - | Implicated along with **miR-155** and **miR21** (see this table) in regulation of Toll-like receptors (TLRs). The dysregulation of these miRNAs may be involved in inflammatory diseases and cancers. [7] |
| **9-5p** | - | - | - | - | - | - | - | - | - | I | I | I | I | I | I | - | - | - | - | I | - | - | - | - | miR-9 induced by TLR4 activation as well as TLR2 and TLR7/8 agonists and by the proinflammatory cytokines (TNF-α and IL-1β, but not by IFN-γ). [8] It has been shown however that miR-9 expression is reduced in some cancers. [9] |
| **155-5p** | I | I | I | I | I | I | - | - | - | I | - | I | - | - | - | I | I | I | I | I | I | - | - | - | miR-155 expression inhibits malignant growth *in vivo*. [10] *see also* **miR-147a.** |
| **135b-5p** | I | I | I | I | I | I | - | - | - | - | I | I | - | - | - | - | - | - | I | - | I | - | - | - | miR-135b is reported to be a is an oncogenic microRNA that enhances cancer cell invasive and migratory abilities in vitro and promotes cancer metastasis in vivo. [11] Other studies demonstrate that miR135b is involved in the regulation of inflammation. miR-135b expression in inflammation is regulated by IL-1R1 in a regulatory feedback mechanism to resolve inflammation. [12] |
| **298** | I | I | I | I | I | I | - | I | - | - | - | - | - | - | - | - | - | - | I | I | I | - | - | - | miR-298-5p and miR-296-3p are causally involved in the higher resistance of mammalian pancreatic α cells to cytokine-induced apoptosis. [13] |
| **19b-3p** | - | I | - | I | I | I | - | - | - | - | - | - | - | - | - | - | - | - | - | - | - | - | - | - | miR-19b-3p promotes colon cancer proliferation and chemoresistance. [14] |
| **183-5p** | I | - | I | I | I | I | - | - | - | - | - | - | - | - | - | - | - | - | - | - | - | - | - | - | The miR-183/-96/-182 polycistronic miRNA cluster is up-regulated in most breast cancers and increases cell proliferation and migration. [15] miR-183 is negatively regulated by transcription factor GATA3. [16] |
| **363-3p** | I | - | I | I | I | I | - | - | - | - | - | - | - | - | - | - | - | - | - | - | - | - | - | - | miR-363 may regulate endothelial cell properties and their communication with hematopoietic precursor cells. [17] Overexpression of miR-363 suppresses the tumourigenicity of colorectal cancer cells. [18] |
| **210-3p** | I | - | I | I | I | I | - | I | - | - | I | - | - | - | - | - | - | - | - | - | - | - | - | - | miR-210 has been identified as a major miRNA induced under hypoxia and generally exhibits oncogenic properties, as it is frequently elevated in several cancers including breast, lung, head and neck, pancreatic cancer, or glioblastoma. [19,20] Oncogenic miR-200-3p also promotes prostate cancer cell epithelial-mesenchymal transition and bone metastasis. [21] |
| **214-3p** | I | - | I | I | I | I | I | I | I | I | I | I | - | - | - | - | - | - | - | - | - | - | - | - | miR-214 is predicted to target two activating protein 2 transcription factors, bringing about downstream effects on a number of genes regulating vital cell cycle processes, such as apoptosis, proliferation and angiogenesis. [22] Upregulation of miR-214 in Hela cells reduced cell growth. [23] Elevated expression in pancreatic cancer may inhibit chemotherapy effectiveness. [24] |
| **149-5p** | I | I | I | I | I | I | - | I | I | - | - | - | - | - | - | - | - | - | - | - | - | - | - | - | Tumor suppressor activity associated with the regulation of apoptosis. [25] Expression of miR-149 is inversely associated with inflammation. miR-149 has regulatory effects on TNF-α, IL-1ß and IL-6. [26] |
| **18b-5p** | I | I | I | I | I | I | - | - | - | - | - | - | - | - | - | - | - | - | - | - | - | - | - | - | miR-18b-5p serves as a tumor suppressor in melanoma. [27] Overexpression of miR-18b identifies mantle cell lymphoma patients with poor prognosis [28] and accelerates hepatocellular carcinoma cell proliferation and loss of cell adhesion ability. [29] |
| **19a-3p** | I | I | I | I | I | I | - | - | - | - | - | - | - | - | - | - | - | - | - | - | - | - | - | - | Downregulation of miR-19a-3p promotes invasion, migration and bone metastasis in prostate cancer. [30] miR-19a-3p promotes tumor metastasis and chemoresistance in hepatocellular carcinoma. [31] |
| **20a-5p** | I | I | I | I | I | I | - | - | - | - | - | - | - | - | - | - | - | - | - | - | - | - | - | - | miR-20a-5p promotes colorectal cancer invasion and metastasis. [32] miR-20a-5p suppresses tumor proliferation in neuroblastoma. [33] |
| **20b-5p** | I | I | I | I | I | I | - | - | - | - | - | - | - | - | - | - | - | - | - | - | - | - | - | - | Low miR-20b-5p expression in peripheral blood mononuclear cells predicts poor overall survival in chronic lymphocytic leukemia patients. [34] |
| **325** | I | I | I | I | I | I | - | D | - | - | - | - | - | - | - | - | - | - | - | - | - | - | - | - | miR-325 regulates autophagic program. [35] miR-325 functions as a tumor suppressor in hepatocellular carcinoma. [36] |
| **21-5p** | - | - | - | I | I | I | - | - | - | - | - | - | - | - | - | - | - | - | - | - | - | - | - | - | A number of targets for miR-21 have been experimentally validated and most of them are tumor suppressors. miR-21 may work in conjunction with miR-34a. [37] see also miR-34a. |
| **98-5p** | - | - | - | I | I | I | - | D | - | - | D | - | - | - | - | - | - | - | - | - | - | - | - | - | Downregulated miR-98-5p promotes pancreatic ductal adenocarcinoma proliferation and metastasis. [38] |
| **187-3p** | - | - | - | I | I | I | - | I | - | - | I | - | - | - | - | - | - | - | - | - | - | - | - | - | miR-187-3p inhibits the metastasis and epithelial-mesenchymal transition of hepatocellular carcinoma. [39] miR-187 is significantly downregulated in prostate cancer. [40] |
| **409-3p** | - | D | - | D | D | D | - | D | - | - | D | - | - | D | - | - | - | - | - | D | - | - | - | - | Downregulation of miR-409-3p promotes aggressiveness and metastasis in colorectal cancer. [41] miR-409-3p is upregulated in breast cancer and its downregulation inhibits cancer development. [42] miR-409-3p/-5p promotes tumorigenesis and metastasis of human prostate cancer. [43] |
| **146a-5p** | D | D | D | D | D | D | - | - | - | - | - | - | - | D | - | - | - | - | - | D | - | - | - | - | miR-146a-5p functions as a tumor suppressor in various types of cancer. [44–47] |
| **379-5p** | D | D | D | D | D | D | - | - | - | - | D | - | D | D | D | - | - | - | - | D | - | - | - | - | miR-379/miR-656 cluster is a tumor suppressor locus involved in multiple cancers, especially in glioblastoma multiforme. [48] miR-374-5p, miR-379-5p, and miR-503-5p regulate proliferation and hypertrophic differentiation of hrowth plate chondrocytes in male rats. [49] |
| **335-5p** | D | D | D | D | D | D | - | D | - | - | D | - | D | D | D | - | - | - | - | D | - | - | - | - | miR-335-5p was identified as a cancer suppressor and found to be downregulated in gastric cancer and involved in tumorigenesis. [50,51] Overexpression of miR-335-5p promotes osteogenic differentiation [52] and bone formation and regeneration in mice. [53] |
| **34a-5p** | D | D | D | D | D | D | - | I | - | - | - | - | - | - | - | - | D | - | - | D | - | - | - | - | miR-34a is a part of the p53 tumor suppressor network. It is hypothesized that miR-34 dysregulation is involved in the development of inflammation and some cancers. [54,55] |
| **132-3p** | D | D | D | D | D | D | - | - | - | - | - | - | - | - | - | - | D | - | - | D | - | - | D | - | miR-132 is extensively involved in the modulation inflammation. One putative target being p300. Down regulation of p300 inhibits expression of IFN-β, ISG15, IL-1β and IL6. [56] In autoimmune rheumatoid arthritis low plasma levels of miR-132 are observed suggesting a dysregulation of inflammation. [57] |
| **181a-5p** | - | D | - | - | - | D | - | I | - | - | I | - | - | - | - | - | - | - | - | D | - | D | D | D | miR-181a-5p inhibits cancer cell proliferation, migration, invasion and angiogenesis via downregulation of matrix metalloproteinase-14 (MMP-14) in human breast and colon cancers [58] or targeting Kras in non-small cell lung cancer A549 cells [59]. In contrast, miR-181a-5p is upregulated in gastric cancer and correlated with invasion [60]; miR-181a-5p facilitates cell proliferation in acute lymphoblastic leukemia by activating Wnt-signaling pathway [61]. |
| **31-5p** | - | - | - | D | D | D | - | - | - | - | - | - | - | - | - | - | - | - | - | D | - | - | D | - | Increased expression of miR-31-5p inhibits cell proliferation, migration, and invasion in HepG2 hepatocellular carcinoma cell line. [62] miR-31-5p/3p associate with time to progression in wild-type RAS metastatic colorectal cancer and are promising biomarkers of cetuximab response. [63] |
| **150-5p** | D | D | D | D | D | D | - | D | - | - | D | - | - | - | - | - | - | - | - | - | - | - | - | - | MiR-150 promotes cellular metastasis in non-small cell lung cancer. [64] Circulating microRNA-150-5p predicts advanced heart failure. [65] |
| **451a** | D | D | D | D | D | D | - | I | - | - | I | - | - | - | - | - | - | - | - | - | - | - | - | - | miR-451 is widely dysregulated in human cancers and plays a critical role in tumorigenesis and tumor progression. [66] miR-451a suppresses cancer cell migration and invasion in hypopharyngeal squamous cell carcinoma. [67] |
| **223-3p** | D | D | D | D | D | D | - | D | - | - | D | - | - | - | - | - | - | - | - | - | - | - | - | - | MiR-223-3p overexpression inhibits cell proliferation and migration by regulating inflammation-associated cytokines in glioblastomas. [68] miR-223-3p Inhibits Human Osteosarcoma Metastasis and Progression. [69] miR-223-3p is upregulated in prostate cancer tissues and promoting the biological behavior of prostate cancer. [70] |
| **26a-5p** | D | D | D | D | D | D | - | D | - | - | D | - | - | - | - | - | - | - | - | - | - | - | - | - | Tumour-suppressive miRNA-26a-5p and miR-26b-5p inhibit cell aggressiveness in bladder cancer. [71] Overexpression of miR-26b-5p regulates the cell cycle in GC-2 cells. [72] |
| **26b-5p** | D | D | D | D | D | D | - | D | - | - | D | - | - | - | - | - | - | - | - | - | - | - | - | - |  |
| **27a-3p** | - | D | - | - | D | - | - | I | - | - | - | - | - | I | - | - | - | - | - | D | - | - | D | - | miR-27a and -27b are implicated in cellular differentiation and are activators of the Wnt signaling pathway. [73] miR-27a has been identified as one of three miRNAS (along with miR-96 and miR-182) which directly target FOXO1 and regulate its endogenous expression. Suppression of miR-27a results in a FOXO1 protein increase and a consequent cell number decrease. [74] The FOXO transcription factor family—which is central to the integration of growth factor signaling, oxidative stress and inflammation may modulate the magnitude of an immune response. FOXO transcription factors may guide T cell differentiation and function in a context-driven manner. [75] |
| **27b-3p** | - | D | - | - | D | - | - | I | - | - | D | - | - | I | - | - | - | - | - | D | - | - | D | - |  |
| **99b-5p** | D | D | D | - | D | - | - | - | - | - | - | - | - | I | - | - | D | - | - | D | - | - | D | - | MiR-99b-5p and miR-203a-3p Function as Tumor Suppressors by Targeting IGF-1R in Gastric Cancer. [76] Low miR-99b-5p expression may correlate with tumor progression in tyrosine kinase inhibitor treated clear cell Renal Cell Carcinoma. [77] |
| **23b-3p** | - | D | - | D | D | D | - | D | - | - | D | - | - | - | - | - | - | - | - | I | - | - | I | - | MicroRNA-23b Functions as a Tumor Suppressor by Regulating Zeb1 in Bladder Cancer. [78] Downregulated miR-23b-3p expression acts as a predictor of hepatocellular carcinoma progression. [79] miR-23b-3p can target either tumor-suppressor genes or oncogenes in different types of tumors; its net biological effect can be tumor-specific. [80] |
| **let-7e-5p** | - | D | - | - | D | - | - | I | - | - | - | - | - | I | - | - | - | - | - | - | - | - | - | - | The lethal-7 (let-7) gene and miRNA were one of the first two known miRNAs (the other one is lin-4) in C. elegans and humans. [81] Evidence suggests that a major function of let-7 genes may be to promote terminal differentiation and apoptosis in development and to act as a 'cancer suppressor'. Let-7 is implicated in the regulation of IL-6, IL-10, IL-13 and a negative regulator of TLR4. [82] |
| **let-7c-5p** | - | D | - | - | D | - | - | I | - | - | I | - | - | - | - | - | - | - | - | - | - | - | - | - |  |
| **let-7a-5p** | - | D | - | - | D | - | - | - | - | - | D | - | - | - | - | - | - | - | - | - | - | - | - | - |  |

**References:**

1. Greither T, Grochola LF, Udelnow A, Lautenschläger C, Würl P, Taubert H. Elevated expression of microRNAs 155, 203, 210 and 222 in pancreatic tumors is associated with poorer survival. Int J Cancer. 2010;126:73-80. 10.1002/ijc.24687

2. Tang X, Tian X, Zhang Y, Wu W, Tian J, Rui K, Tong J, Lu L, Xu H, Wang S. Correlation between the frequency of Th17 cell and the expression of microRNA-206 in patients with dermatomyositis. Clin Dev Immunol. 2013;2013:345347. 10.1155/2013/345347

3. Lin SL, Chang DC, Chang-Lin S, Lin CH, Wu DT, Chen DT, Ying SY. Mir-302 reprograms human skin cancer cells into a pluripotent ES-cell-like state. RNA. 2008;14:2115-2124. 10.1261/rna.1162708

4. Barroso-del Jesus A, Lucena-Aguilar G, Menendez P. The miR-302-367 cluster as a potential stemness regulator in ESCs. Cell Cycle. 2009;8:394-398. 10.4161/cc.8.3.7554

5. Lin SL, Chang DC, Ying SY, Leu D, Wu DT. MicroRNA miR-302 inhibits the tumorigenecity of human pluripotent stem cells by coordinate suppression of the CDK2 and CDK4/6 cell cycle pathways. Cancer Res. 2010;70:9473-9482. 10.1158/0008-5472.CAN-10-2746

6. Jiang H, Zhang G, Wu JH, Jiang CP. Diverse roles of miR-29 in cancer (review). Oncol Rep. 2014;31:1509-1516. 10.3892/or.2014.3036

7. Quinn SR, O’Neill LA. A trio of microRNAs that control Toll-like receptor signalling. Int Immunol. 2011;23:421-425. 10.1093/intimm/dxr034

8. Bazzoni F, Rossato M, Fabbri M, Gaudiosi D, Mirolo M, Mori L, Tamassia N, Mantovani A, Cassatella MA, Locati M. Induction and regulatory function of miR-9 in human monocytes and neutrophils exposed to proinflammatory signals. Proc Natl Acad Sci U S A. 2009;106:5282-5287. 10.1073/pnas.0810909106

9. Lehmann U, Hasemeier B, Christgen M, Müller M, Römermann D, Länger F, Kreipe H. Epigenetic inactivation of microRNA gene hsa-mir-9-1 in human breast cancer. J Pathol. 2008;214:17-24. 10.1002/path.2251

10. Babar IA, Cheng CJ, Booth CJ, Liang X, Weidhaas JB, Saltzman WM, Slack FJ. Nanoparticle-based therapy in an in vivo microRNA-155 (miR-155)-dependent mouse model of lymphoma. Proc Natl Acad Sci U S A. 2012;109:E1695-704. 10.1073/pnas.1201516109

11. Lin CW, Chang YL, Chang YC, Lin JC, Chen CC, Pan SH, Wu CT, Chen HY, Yang SC, Hong TM, Yang PC. MicroRNA-135b promotes lung cancer metastasis by regulating multiple targets in the Hippo pathway and LZTS1. Nat Commun. 2013;4:1877. 10.1038/ncomms2876

12. Halappanavar S, Nikota J, Wu D, Williams A, Yauk CL, Stampfli M. IL-1 receptor regulates microRNA-135b expression in a negative feedback mechanism during cigarette smoke-induced inflammation. J Immunol. 2013;190:3679-3686. 10.4049/jimmunol.1202456

13. Barbagallo D, Piro S, Condorelli AG, Mascali LG, Urbano F, Parrinello N, Monello A, Statello L, Ragusa M, Rabuazzo AM, Di Pietro C, Purrello F, Purrello M. miR-296-3p, miR-298-5p and their downstream networks are causally involved in the higher resistance of mammalian pancreatic α cells to cytokine-induced apoptosis as compared to β cells. BMC Genomics. 2013;14:62. 10.1186/1471-2164-14-62

14. Jiang T, Ye L, Han Z, Liu Y, Yang Y, Peng Z, Fan J. miR-19b-3p promotes colon cancer proliferation and oxaliplatin-based chemoresistance by targeting SMAD4: validation by bioinformatics and experimental analyses. J Exp Clin Cancer Res. 2017;36:131. 10.1186/s13046-017-0602-5

15. Li P, Sheng C, Huang L, Zhang H, Huang L, Cheng Z, Zhu Q. MiR-183/-96/-182 cluster is up-regulated in most breast cancers and increases cell proliferation and migration. Breast Cancer Res. 2014;16:473. 10.1186/s13058-014-0473-z

16. Chen C, Xiang H, Peng YL, Peng J, Jiang SW. Mature miR-183, negatively regulated by transcription factor GATA3, promotes 3T3-L1 adipogenesis through inhibition of the canonical Wnt/β-catenin signaling pathway by targeting LRP6. Cell Signal. 2014;26:1155-1165. 10.1016/j.cellsig.2014.02.003

17. Costa A, Afonso J, Osório C, Gomes AL, Caiado F, Valente J, Aguiar SI, Pinto F, Ramirez M, Dias S. miR-363-5p regulates endothelial cell properties and their communication with hematopoietic precursor cells. J Hematol Oncol. 2013;6:87. 10.1186/1756-8722-6-87

18. Tsuji S, Kawasaki Y, Furukawa S, Taniue K, Hayashi T, Okuno M, Hiyoshi M, Kitayama J, Akiyama T. The miR-363-GATA6-Lgr5 pathway is critical for colorectal tumourigenesis. Nat Commun. 2014;5:3150. 10.1038/ncomms4150

19. Bavelloni A, Ramazzotti G, Poli A, Piazzi M, Focaccia E, Blalock W, Faenza I. MiRNA-210: A Current Overview. Anticancer Res. 2017;37:6511-6521. 10.21873/anticanres.12107

20. Qin Q, Furong W, Baosheng L. Multiple functions of hypoxia-regulated miR-210 in cancer. J Exp Clin Cancer Res. 2014;33:50. 10.1186/1756-9966-33-50

21. Ren D, Yang Q, Dai Y, Guo W, Du H, Song L, Peng X. Oncogenic miR-210-3p promotes prostate cancer cell EMT and bone metastasis via NF-κB signaling pathway. Mol Cancer. 2017;16:117. 10.1186/s12943-017-0688-6

22. Bar-Eli M. Searching for the ‘melano-miRs’: miR-214 drives melanoma metastasis. EMBO J. 2011;30:1880-1881. 10.1038/emboj.2011.132

23. Yang Z, Chen S, Luan X, Li Y, Liu M, Li X, Liu T, Tang H. MicroRNA-214 is aberrantly expressed in cervical cancers and inhibits the growth of HeLa cells. IUBMB Life. 2009;61:1075-1082. 10.1002/iub.252

24. Zhang XJ, Ye H, Zeng CW, He B, Zhang H, Chen YQ. Dysregulation of miR-15a and miR-214 in human pancreatic cancer. J Hematol Oncol. 2010;3:46. 10.1186/1756-8722-3-46

25. Lin RJ, Lin YC, Yu AL. miR-149* induces apoptosis by inhibiting Akt1 and E2F1 in human cancer cells. Mol Carcinog. 2010;49:719-727. 10.1002/mc.20647

26. Santini P, Politi L, Vedova PD, Scandurra R, Scotto d’Abusco A. The inflammatory circuitry of miR-149 as a pathological mechanism in osteoarthritis. Rheumatol Int. 2014;34:711-716. 10.1007/s00296-013-2754-8

27. Dar AA, Majid S, Rittsteuer C, de Semir D, Bezrookove V, Tong S, Nosrati M, Sagebiel R, Miller JR, Kashani-Sabet M. The role of miR-18b in MDM2-p53 pathway signaling and melanoma progression. J Natl Cancer Inst. 2013;105:433-442. 10.1093/jnci/djt003

28. Husby S, Ralfkiaer U, Garde C, Zandi R, Ek S, Kolstad A, Jerkeman M, Laurell A, Räty R, Pedersen LB, Pedersen A, Ehinger M, Sundström C, Karjalainen-Lindsberg ML, Delabie J, Clasen-Linde E, Brown P, Cowland JB, Workman CT, Geisler CH, Grønbæk K. miR-18b overexpression identifies mantle cell lymphoma patients with poor outcome and improves the MIPI-B prognosticator. Blood. 2015;125:2669-2677. 10.1182/blood-2014-06-584193

29. Murakami Y, Tamori A, Itami S, Tanahashi T, Toyoda H, Tanaka M, Wu W, Brojigin N, Kaneoka Y, Maeda A, Kumada T, Kawada N, Kubo S, Kuroda M. The expression level of miR-18b in hepatocellular carcinoma is associated with the grade of malignancy and prognosis. BMC Cancer. 2013;13:99. 10.1186/1471-2407-13-99

30. Wa Q, Li L, Lin H, Peng X, Ren D, Huang Y, He P, Huang S. Downregulation of miR‑19a‑3p promotes invasion, migration and bone metastasis via activating TGF‑β signaling in prostate cancer. Oncol Rep. 2018;39:81-90. 10.3892/or.2017.6096

31. Jiang XM, Yu XN, Liu TT, Zhu HR, Shi X, Bilegsaikhan E, Guo HY, Song GQ, Weng SQ, Huang XX, Dong L, Janssen HLA, Shen XZ, Zhu JM. microRNA-19a-3p promotes tumor metastasis and chemoresistance through the PTEN/Akt pathway in hepatocellular carcinoma. Biomed Pharmacother. 2018;105:1147-1154. 10.1016/j.biopha.2018.06.097

32. Cheng D, Zhao S, Tang H, Zhang D, Sun H, Yu F, Jiang W, Yue B, Wang J, Zhang M, Yu Y, Liu X, Sun X, Zhou Z, Qin X, Zhang X, Yan D, Wen Y, Peng Z. MicroRNA-20a-5p promotes colorectal cancer invasion and metastasis by downregulating Smad4. Oncotarget. 2016;7:45199-45213. 10.18632/oncotarget.9900

33. Yu Y, Zhang J, Jin Y, Yang Y, Shi J, Chen F, Han S, Chu P, Lu J, Wang H, Guo Y, Ni X. MiR-20a-5p suppresses tumor proliferation by targeting autophagy-related gene 7 in neuroblastoma. Cancer Cell Int. 2018;18:5. 10.1186/s12935-017-0499-2

34. Papageorgiou SG, Kontos CK, Tsiakanikas P, Stavroulaki G, Bouchla A, Vasilatou D, Bazani E, Lazarakou A, Scorilas A, Pappa V. Elevated miR-20b-5p expression in peripheral blood mononuclear cells: A novel, independent molecular biomarker of favorable prognosis in chronic lymphocytic leukemia. Leuk Res. 2018;70:1-7. 10.1016/j.leukres.2018.04.014

35. Bo L, Su-Ling D, Fang L, Lu-Yu Z, Tao A, Stefan D, Kun W, Pei-Feng L. Autophagic program is regulated by miR-325. Cell Death Differ. 2014;21:967-977. 10.1038/cdd.2014.18

36. Li H, Huang W, Luo R. The microRNA-325 inhibits hepatocellular carcinoma progression by targeting high mobility group box 1. Diagn Pathol. 2015;10:117. 10.1186/s13000-015-0323-z

37. Hashimi ST, Fulcher JA, Chang MH, Gov L, Wang S, Lee B. MicroRNA profiling identifies miR-34a and miR-21 and their target genes JAG1 and WNT1 in the coordinate regulation of dendritic cell differentiation. Blood. 2009;114:404-414. 10.1182/blood-2008-09-179150

38. Fu Y, Liu X, Chen Q, Liu T, Lu C, Yu J, Miao Y, Wei J. Downregulated miR-98-5p promotes PDAC proliferation and metastasis by reversely regulating MAP4K4. J Exp Clin Cancer Res. 2018;37:130. 10.1186/s13046-018-0807-2

39. Dou C, Liu Z, Xu M, Jia Y, Wang Y, Li Q, Yang W, Zheng X, Tu K, Liu Q. miR-187-3p inhibits the metastasis and epithelial-mesenchymal transition of hepatocellular carcinoma by targeting S100A4. Cancer Lett. 2016;381:380-390. 10.1016/j.canlet.2016.08.011

40. Casanova-Salas I, Masiá E, Armiñán A, Calatrava A, Mancarella C, Rubio-Briones J, Scotlandi K, Vicent MJ, López-Guerrero JA. MiR-187 Targets the Androgen-Regulated Gene ALDH1A3 in Prostate Cancer. PLoS One. 2015;10:e0125576. 10.1371/journal.pone.0125576

41. Liu M, Xu A, Yuan X, Zhang Q, Fang T, Wang W, Li C. Downregulation of microRNA-409-3p promotes aggressiveness and metastasis in colorectal cancer: an indication for personalized medicine. J Transl Med. 2015;13:195. 10.1186/s12967-015-0533-x

42. Yu H, Xing H, Han W, Wang Y, Qi T, Song C, Xu Z, Li H, Huang Y. MicroRNA-409-5p is upregulated in breast cancer and its downregulation inhibits cancer development through downstream target of RSU1. Tumour Biol. 2017;39:1010428317701647. 10.1177/1010428317701647

43. Josson S, Gururajan M, Hu P, Shao C, Chu GY, Zhau HE, Liu C, Lao K, Lu CL, Lu YT, Lichterman J, Nandana S, Li Q, Rogatko A, Berel D, Posadas EM, Fazli L, Sareen D, Chung LW. miR-409-3p/-5p promotes tumorigenesis, epithelial-to-mesenchymal transition, and bone metastasis of human prostate cancer. Clin Cancer Res. 2014;20:4636-4646. 10.1158/1078-0432.CCR-14-0305

44. Zhang X, Ye ZH, Liang HW, Ren FH, Li P, Dang YW, Chen G. Down-regulation of miR-146a-5p and its potential targets in hepatocellular carcinoma validated by a TCGA- and GEO-based study. FEBS Open Bio. 2017;7:504-521. 10.1002/2211-5463.12198

45. Wang C, Zhang W, Zhang L, Chen X, Liu F, Zhang J, Guan S, Sun Y, Chen P, Wang D, Un Nesa E, Cheng Y, Yousef GM. miR-146a-5p mediates epithelial-mesenchymal transition of oesophageal squamous cell carcinoma via targeting Notch2. Br J Cancer. 2016;115:1548-1554. 10.1038/bjc.2016.367

46. Li YL, Wang J, Zhang CY, Shen YQ, Wang HM, Ding L, Gu YC, Lou JT, Zhao XT, Ma ZL, Jin YX. MiR-146a-5p inhibits cell proliferation and cell cycle progression in NSCLC cell lines by targeting CCND1 and CCND2. Oncotarget. 2016;7:59287-59298. 10.18632/oncotarget.11040

47. Xu B, Huang Y, Niu X, Tao T, Jiang L, Tong N, Chen S, Liu N, Zhu W, Chen M. Hsa-miR-146a-5p modulates androgen-independent prostate cancer cells apoptosis by targeting ROCK1. Prostate. 2015;75:1896-1903. 10.1002/pros.23068

48. Laddha SV, Nayak S, Paul D, Reddy R, Sharma C, Jha P, Hariharan M, Agrawal A, Chowdhury S, Sarkar C, Mukhopadhyay A. Genome-wide analysis reveals downregulation of miR-379/miR-656 cluster in human cancers. Biol Direct. 2013;8:10. 10.1186/1745-6150-8-10

49. Jee YH, Wang J, Yue S, Jennings M, Clokie SJ, Nilsson O, Lui JC, Baron J. mir-374-5p, mir-379-5p, and mir-503-5p Regulate Proliferation and Hypertrophic Differentiation of Growth Plate Chondrocytes in Male Rats. Endocrinology. 2018;159:1469-1478. 10.1210/en.2017-00780

50. Sandoval-Bórquez A, Polakovicova I, Carrasco-Véliz N, Lobos-González L, Riquelme I, Carrasco-Avino G, Bizama C, Norero E, Owen GI, Roa JC, Corvalán AH. MicroRNA-335-5p is a potential suppressor of metastasis and invasion in gastric cancer. Clin Epigenetics. 2017;9:114. 10.1186/s13148-017-0413-8

51. Li H, Xie S, Liu M, Chen Z, Liu X, Wang L, Li D, Zhou Y. The clinical significance of downregulation of mir-124-3p, mir-146a-5p, mir-155-5p and mir-335-5p in gastric cancer tumorigenesis. Int J Oncol. 2014;45:197-208. 10.3892/ijo.2014.2415

52. Zhang J, Tu Q, Bonewald LF, He X, Stein G, Lian J, Chen J. Effects of miR-335-5p in modulating osteogenic differentiation by specifically downregulating Wnt antagonist DKK1. J Bone Miner Res. 2011;26:1953-1963. 10.1002/jbmr.377

53. Zhang L, Tang Y, Zhu X, Tu T, Sui L, Han Q, Yu L, Meng S, Zheng L, Valverde P, Tang J, Murray D, Zhou X, Drissi H, Dard MM, Tu Q, Chen J. Overexpression of MiR-335-5p Promotes Bone Formation and Regeneration in Mice. J Bone Miner Res. 2017;32:2466-2475. 10.1002/jbmr.3230

54. Chen QR, Yu LR, Tsang P, Wei JS, Song YK, Cheuk A, Chung JY, Hewitt SM, Veenstra TD, Khan J. Systematic proteome analysis identifies transcription factor YY1 as a direct target of miR-34a. J Proteome Res. 2011;10:479-487. 10.1021/pr1006697

55. He L, He X, Lim LP, de Stanchina E, Xuan Z, Liang Y, Xue W, Zender L, Magnus J, Ridzon D, Jackson AL, Linsley PS, Chen C, Lowe SW, Cleary MA, Hannon GJ. A microRNA component of the p53 tumour suppressor network. Nature. 2007;447:1130-1134. 10.1038/nature05939

56. Lagos D, Pollara G, Henderson S, Gratrix F, Fabani M, Milne RS, Gotch F, Boshoff C. miR-132 regulates antiviral innate immunity through suppression of the p300 transcriptional co-activator. Nat Cell Biol. 2010;12:513-519. 10.1038/ncb2054

57. Murata K, Yoshitomi H, Tanida S, Ishikawa M, Nishitani K, Ito H, Nakamura T. Plasma and synovial fluid microRNAs as potential biomarkers of rheumatoid arthritis and osteoarthritis. Arthritis Res Ther. 2010;12:R86. 10.1186/ar3013

58. Li Y, Kuscu C, Banach A, Zhang Q, Pulkoski-Gross A, Kim D, Liu J, Roth E, Li E, Shroyer KR, Denoya PI, Zhu X, Chen L, Cao J. miR-181a-5p Inhibits Cancer Cell Migration and Angiogenesis via Downregulation of Matrix Metalloproteinase-14. Cancer Res. 2015;75:2674-2685. 10.1158/0008-5472.CAN-14-2875

59. Ma Z, Qiu X, Wang D, Li Y, Zhang B, Yuan T, Wei J, Zhao B, Zhao X, Lou J, Jin Y, Jin Y. MiR-181a-5p inhibits cell proliferation and migration by targeting Kras in non-small cell lung cancer A549 cells. Acta Biochim Biophys Sin (Shanghai). 2015;47:630-638. 10.1093/abbs/gmv054

60. Chen G, Shen ZL, Wang L, Lv CY, Huang XE, Zhou RP. Hsa-miR-181a-5p expression and effects on cell proliferation in gastric cancer. Asian Pac J Cancer Prev. 2013;14:3871-3875. 10.7314/APJCP.2013.14.6.3871

61. Lyu X, Li J, Yun X, Huang R, Deng X, Wang Y, Chen Y, Xiao G. miR-181a-5p, an inducer of Wnt-signaling, facilitates cell proliferation in acute lymphoblastic leukemia. Oncol Rep. 2017;37:1469-1476. 10.3892/or.2017.5425

62. Zhao G, Han C, Zhang Z, Wang L, Xu J. Increased expression of microRNA-31-5p inhibits cell proliferation, migration, and invasion via regulating Sp1 transcription factor in HepG2 hepatocellular carcinoma cell line. Biochem Biophys Res Commun. 2017;490:371-377. 10.1016/j.bbrc.2017.06.050

63. Mlcochova J, Faltejskova-Vychytilova P, Ferracin M, Zagatti B, Radova L, Svoboda M, Nemecek R, John S, Kiss I, Vyzula R, Negrini M, Slaby O. MicroRNA expression profiling identifies miR-31-5p/3p as associated with time to progression in wild-type RAS metastatic colorectal cancer treated with cetuximab. Oncotarget. 2015;6:38695-38704. 10.18632/oncotarget.5735

64. Li H, Ouyang R, Wang Z, Zhou W, Chen H, Jiang Y, Zhang Y, Li H, Liao M, Wang W, Ye M, Ding Z, Feng X, Liu J, Zhang B. MiR-150 promotes cellular metastasis in non-small cell lung cancer by targeting FOXO4. Sci Rep. 2016;6:39001. 10.1038/srep39001

65. Scrutinio D, Conserva F, Passantino A, Iacoviello M, Lagioia R, Gesualdo L. Circulating microRNA-150-5p as a novel biomarker for advanced heart failure: A genome-wide prospective study. J Heart Lung Transplant. 2017;36:616-624. 10.1016/j.healun.2017.02.008

66. Pan X, Wang R, Wang ZX. The potential role of miR-451 in cancer diagnosis, prognosis, and therapy. Mol Cancer Ther. 2013;12:1153-1162. 10.1158/1535-7163.MCT-12-0802

67. Fukumoto I, Kinoshita T, Hanazawa T, Kikkawa N, Chiyomaru T, Enokida H, Yamamoto N, Goto Y, Nishikawa R, Nakagawa M, Okamoto Y, Seki N. Identification of tumour suppressive microRNA-451a in hypopharyngeal squamous cell carcinoma based on microRNA expression signature. Br J Cancer. 2014;111:386-394. 10.1038/bjc.2014.293

68. Ding Q, Shen L, Nie X, Lu B, Pan X, Su Z, Yan A, Yan R, Zhou Y, Li L, Xu J. MiR-223-3p overexpression inhibits cell proliferation and migration by regulating inflammation-associated cytokines in glioblastomas. Pathol Res Pract. 2018;214:1330-1339. 10.1016/j.prp.2018.05.012

69. Ji Q, Xu X, Song Q, Xu Y, Tai Y, Goodman SB, Bi W, Xu M, Jiao S, Maloney WJ, Wang Y. miR-223-3p Inhibits Human Osteosarcoma Metastasis and Progression by Directly Targeting CDH6. Mol Ther. 2018;26:1299-1312. 10.1016/j.ymthe.2018.03.009

70. Wei Y, Yang J, Yi L, Wang Y, Dong Z, Liu Z, Ou-yang S, Wu H, Zhong Z, Yin Z, Zhou K, Gao Y, Yan B, Wang Z. MiR-223-3p targeting SEPT6 promotes the biological behavior of prostate cancer. Sci Rep. 2014;4:7546. 10.1038/srep07546

71. Miyamoto K, Seki N, Matsushita R, Yonemori M, Yoshino H, Nakagawa M, Enokida H. Tumour-suppressive miRNA-26a-5p and miR-26b-5p inhibit cell aggressiveness by regulating PLOD2 in bladder cancer. Br J Cancer. 2016;115:354-363. 10.1038/bjc.2016.179

72. Liu Y, Liu WB, Liu KJ, Ao L, Cao J, Zhong JL, Liu JY. Overexpression of miR-26b-5p regulates the cell cycle by targeting CCND2 in GC-2 cells under exposure to extremely low frequency electromagnetic fields. Cell Cycle. 2016;15:357-367. 10.1080/15384101.2015.1120924

73. Wang T, Xu Z. miR-27 promotes osteoblast differentiation by modulating Wnt signaling. Biochem Biophys Res Commun. 2010;402:186-189. 10.1016/j.bbrc.2010.08.031

74. Guttilla IK, White BA. Coordinate regulation of FOXO1 by miR-27a, miR-96, and miR-182 in breast cancer cells. J Biol Chem. 2009;284:23204-23216. 10.1074/jbc.M109.031427

75. Hedrick SM, Hess Michelini R, Doedens AL, Goldrath AW, Stone EL. FOXO transcription factors throughout T cell biology. Nat Rev Immunol. 2012;12:649-661. 10.1038/nri3278

76. Wang Z, Zhao Z, Yang Y, Luo M, Zhang M, Wang X, Liu L, Hou N, Guo Q, Song T, Guo B, Huang C. MiR-99b-5p and miR-203a-3p Function as Tumor Suppressors by Targeting IGF-1R in Gastric Cancer. Sci Rep. 2018;8:10119. 10.1038/s41598-018-27583-y

77. Lukamowicz-Rajska M, Mittmann C, Prummer M, Zhong Q, Bedke J, Hennenlotter J, Stenzl A, Mischo A, Bihr S, Schmidinger M, Vogl U, Blume I, Karlo C, Schraml P, Moch H. MiR-99b-5p expression and response to tyrosine kinase inhibitor treatment in clear cell renal cell carcinoma patients. Oncotarget. 2016;7:78433-78447. 10.18632/oncotarget.12618

78. Majid S, Dar AA, Saini S, Deng G, Chang I, Greene K, Tanaka Y, Dahiya R, Yamamura S. MicroRNA-23b functions as a tumor suppressor by regulating Zeb1 in bladder cancer. PLoS One. 2013;8:e67686. 10.1371/journal.pone.0067686

79. He RQ, Wu PR, Xiang XL, Yang X, Liang HW, Qiu XH, Yang LH, Peng ZG, Chen G. Downregulated miR-23b-3p expression acts as a predictor of hepatocellular carcinoma progression: A study based on public data and RT-qPCR verification. Int J Mol Med. 2018;41:2813-2831. 10.3892/ijmm.2018.3513

80. Grossi I, Salvi A, Baiocchi G, Portolani N, De Petro G. Functional Role of microRNA-23b-3p in Cancer Biology. Microrna. 2018;7:156-166. 10.2174/2211536607666180629155025

81. Pasquinelli AE, Reinhart BJ, Slack F, Martindale MQ, Kuroda MI, Maller B, Hayward DC, Ball EE, Degnan B, Müller P, Spring J, Srinivasan A, Fishman M, Finnerty J, Corbo J, Levine M, Leahy P, Davidson E, Ruvkun G. Conservation of the sequence and temporal expression of let-7 heterochronic regulatory RNA. Nature. 2000;408:86-89. 10.1038/35040556

82. Barh D, Malhotra R, Ravi B, Sindhurani P. MicroRNA let-7: an emerging next-generation cancer therapeutic. Curr Oncol. 2010;17:70-80. 10.3747/co.v17i1.356
